# Supplementary material for: A homozygous synonymous NOP58 variant causes a neurodevelopmental disorder by impairing maturation of pre-ribosomal RNAs
Source: HGG Adv. 2025 Dec 11;7(1):100557. doi: 10.1016/j.xhgg.2025.100557 (PMC12800697; doi:10.1016/j.xhgg.2025.100557)
Supplement: Document S1. Figures S1 and S2, Tables S1 and S2, and supplemental methods [file mmc1.pdf]

HGGA, Volume ■ ■

Supplemental information

A homozygous synonymous *NOP58* variant causes a neurodevelopmental disorder by impairing maturation of pre-ribosomal RNAs

Loisa D. Bonde, Tess Holling, Malik Alawi, Ahmed A. El Beheiry, Zabih Mir Hassani, François Bachand, Ibrahim M. Abdelrazek, and Kerstin Kutsche

SUPPLEMENTAL INFORMATION

SUPPLEMENTAL FIGURES AND LEGENDS

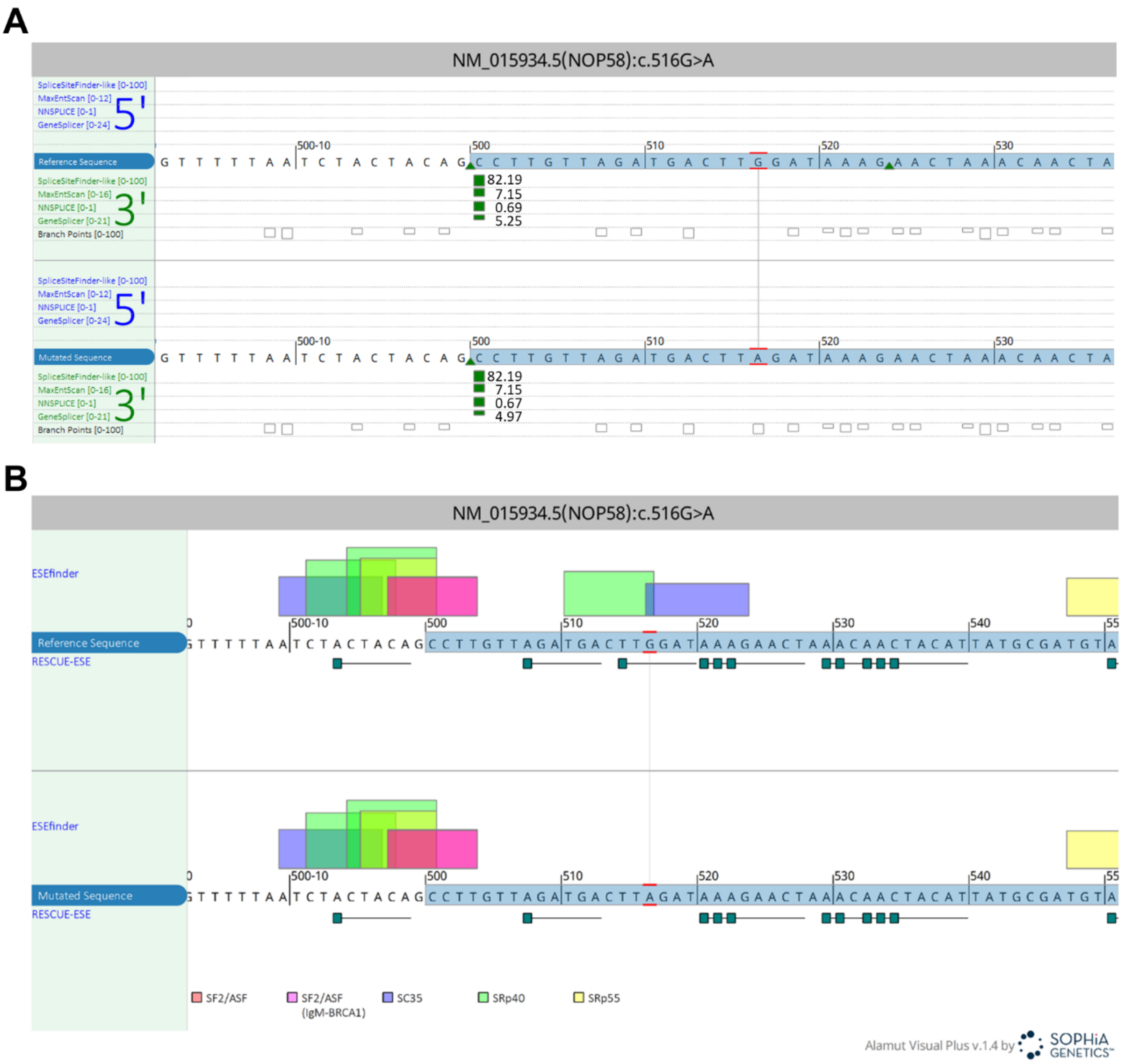

Figure S1. Splice site and exonic splicing enhancer predictions for the homozygous synonymous variant c.516G>A in *NOP58*.

(A-B) Screenshots from the splicing module of Alamut Visual Plus (v1.4; SOPHIA GENETICS) show partial sequences of the intron 6-exon 7 boundary of *NOP58* (NM_015934.5). The reference sequence is displayed in the upper panel and the sequence with the variant c.516G>A in the lower panel. The exonic sequence is highlighted in blue. **(A)** Splice sites were predicted using the programs SpliceSiteFinder-like,[1,2] NNSPLICE 0.9 version,[3] MaxEntScan,[4] and GeneSplicer.[5] The predicted splice acceptor sites are indicated by a green rectangle within the sequence; scores are given (see also Table S2) and indicated as green boxes below the first nucleotide of exon 7. **(B)** Exonic splicing enhancers (ESE) were predicted using the programs ESEfinder[6] and RESCUE-ESE.[7] ESE hits from ESEfinder are displayed as colored boxes above each sequence. The height of each box indicates the score value, while its width and position show the length and position of the motif in the sequence. Predicted RESCUE-ESE hexamers are shown beneath each sequence as petrol boxes, with a line indicating the length of the motif.

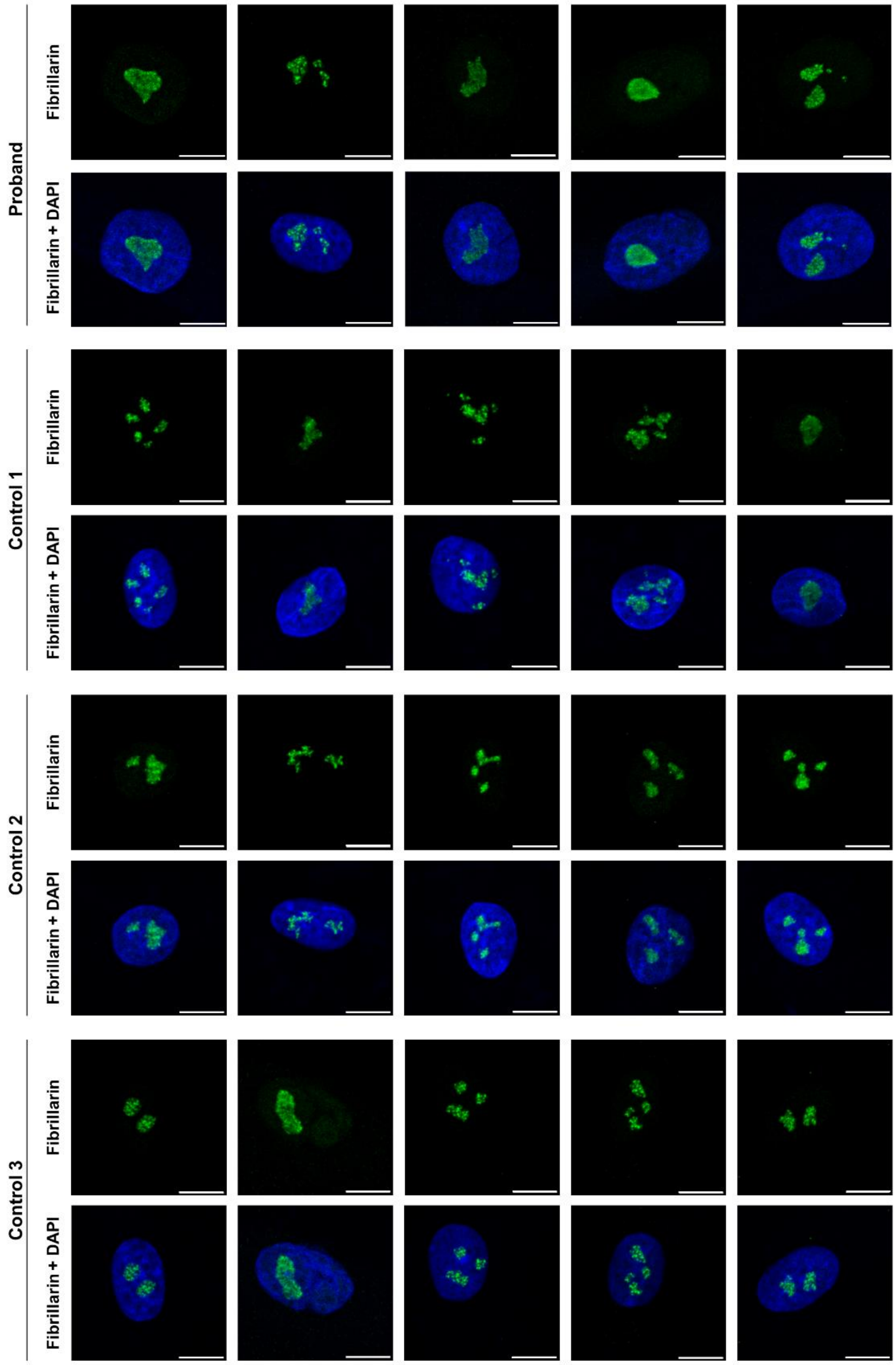

Figure S2. Representative immunofluorescence images of fibrillarin staining in proband and control cells.

Immunofluorescence analysis using an anti-fibrillarin antibody followed by anti-mouse Alexa Fluor 488-conjugated secondary antibody (green) to stain nucleoli in proband and control fibroblasts. Nuclear DNA was stained with DAPI (blue). Fibroblasts were seeded on coverslips and cultivated under basal conditions. Cells were imaged by confocal fluorescence microscopy. Representative images of fibroblasts of the proband (top) and fibroblasts from three controls are shown. Scale bar, 10 µm.

SUPPLEMENTAL TABLES

Table S1. Sequence of oligonucleotides used in this work.

***NOP58* primer sequences for variant validation**			
**Template**	**Exon**	**Direction**	**Sequence (5′ → 3′)**
DNA	7	forward	TGCCTGTCTGCTGGGATTAC
		reverse	CAAAAACCCTGGGAATCTTGC

***NOP58* primer sequences for RT-PCR**				
**Template**	**Name**	**Exon**	**Direction**	**Sequence (5′ → 3′)**
cDNA	c5F	5	forward	CGTGAAATGGCAGCTATGTGTC
	c8R	8	reverse	TCAGAAAGCTTGGCAGAGGC
	c7R	7	reverse	TCCTAATTCAGGGAAATGCCAGCC

***NOP58* primer sequences for RT-qPCR**				
**Template**	**Name**	**Exon**	**Direction**	**Sequence (5′ → 3′)**
cDNA	5F	5	forward	CTCAGTTGTATCCATAGTCC
	6R	6	reverse	CTTTATCAGCGCTAAACTTC
	11F	11	forward	GAGAACTTTGGAAGACAGAG
	13R	13	reverse	TTTAGAACAGGTTGGAAGTG
	6F	6	forward	CGCTGATAAAGTAGACACAA
	7R	7	reverse	TTCCTAATTCAGGGAAATGC
	7F	7	forward	CTACATTATGCGATGTAGAG
	8R	8	reverse	GAGGCATAGTTCTTCCTATC

**snoRNA primer sequences for RT-qPCR**			
**Template**	**Target**	**Direction**	**Sequence (5′ → 3′)**
cDNA	*SNORD14A* (*U14A*) NR_000022.1	forward	GATTGGTTGCCAGACATTCG
		reverse	CACTCAGACATCCAAGGAAG
	SNORD93 (*HBII-336*) NR_003075.1	forward	GCCAAGGATGAGAACTCTAATCTGA
		reverse	GGCCTCAGGTAAATCCTTTAATCCA
	SNORD125 NR_003686.1	forward	CCCTCCTGATGATTCTTCTTCC
		reverse	GTCAACTTCTTAGAGGCTCAGTT
	SNORD18A (*U18A*) NR_002441.1	forward	TAGTGATGAAATTCCACTTC
		reverse	CATCAGAACATCCGAGAAAA
	SNORD91B (*HBII-296B*) NR_003073.1	forward	GTCTGAACCTGTCTGAAGCATCC
		reverse	AAGCCTCAGTATCACACAGAAGT
	SNORD3A (*U3*) NR_006880.1	forward	TAGCAGAGGTGTGCAAGGAGG
		reverse	GCAGTTGCAGCCAAGCAACG
	SNORA30 (*ACA30*) NR_002966.1	forward	GCACTTTCACAGTTCCTTCC
		reverse	CAGGGCAAGAATACAATCAAGG

**rRNA primer sequences for RT-qPCR**			
cDNA	r01-F	forward	CGTGCGTGTCAGGCGTTCTC
	r01-R	reverse	GGTCCCGTTCGCCACGAAC
	r02-F	forward	CAGAGCAGCTCCCTCGCTG
	r02-R	reverse	GGAGGCGGGAACCGAAGAAG
	r2-F	forward	GTGGGGGCTTTACCCGGC
	r2-R	reverse	CCCAAGAGGAGAGGGGGTTG
	r3′-F	forward	CTCTCCCGTCGCCTCTCC
	r3′-R	reverse	CAGAGCAGCTCCCTCGCTG
	rA0-F	forward	GGGGGAGAAGCGAGGGTTCC
	rA0-R	reverse	AGTCACGCGCCGGACAGAG
	r1-F	forward	CCGTCGTCCTCCTCGCTTG

	r1-R	reverse	GAGCGAGCGACCAAAGGAACC
	rC-F	forward	GAGCGAGCGACCAAAGGAACC
cDNA	rC-R	reverse	GCCGGGTAAAGCCCCCAC
	r18S-F	forward	GTCCCTGCCCTTTGTACACAC
	rE-R	reverse	CCGTCTCCCTCCCGAGTTC
	r3-R	reverse	CCTCCGGGCTCCGTTAATG
	r18S-total-R	reverse	GATCCTTCCGCAGGTTCACC

Exon numbering is given according to the *NOP58* reference sequence NM_015934.5.

Table S2. *In silico* pathogenicity and splice site predictions and minor allele frequency of the *NOP58* variant.

Genomic position on chromosome 2 (hg38; NC_000002.12)	Nucleotide and predicted amino acid change (NM_015934.5)	Exon	Variant description after transcript analysis	gnomAD (v4.1.0)	RGC Million Exome	CADD (>20)	Splice site predictions							
							SpliceSite Finder-like (range 0-100)		NNSPLICE (range 0-1)		MaxEntScan (range 0-16)		GeneSplicer (range 0-21)	
							WT	Var	WT	Var	WT	Var	WT	Var
g.202290339G>A	c.516G>A; p.Leu172=	7	r.500_634del; p.(Ser167_Gly212delinsCys)	absent	absent	17.25	82.19	82.19	0.69	0.67	7.15	7.15	5.25	4.97

Worldwide allele frequency of *NOP58* variant in the gnomAD database v4.1.0 [8] and the Regeneron Genetics Center (RGC) Million Exome data[9] is given. The functional impact of the homozygous *NOP58* variants was predicted by the Combined Annotation Dependent Depletion (CADD) tool. CADD is a framework that integrates multiple annotations in one metric by contrasting variants that survived natural selection with simulated mutations. Reported CADD scores are phred-like rank scores based on the rank of that variant's score among all possible single nucleotide variants of hg19, with 10 corresponding to the top 10%, 20 at the top 1%, and 30 at the top 0.1%. The larger the score the more likely the variant has deleterious effects; the score range observed here is strongly supportive of pathogenicity, with all observed variants ranking above ~99% of all variants in a typical genome and scoring similarly to variants reported in ClinVar as pathogenic (~85% of which scores >15).[10] Acceptor splice site prediction scores were predicted for wild-type and mutated sequences using Alamut Visual Plus (v1.4; SOPHIA GENETICS) which implements the following splice site prediction algorithms: SpliceSiteFinder-like,[1,2] NNSPLICE 0.9 version,[3] MaxEntScan,[4] and GeneSplicer.[5] High scores indicate strong splice sites. Var, variant; WT, wildtype.

SUPPLEMENTAL METHODS

Trio whole-exome sequencing and variant filtering

Genomic DNA was extracted from leukocytes of the proband and his parents by standard procedures. Trio whole-exome sequencing (WES) was performed on genomic DNA samples of the proband and his parents. Exome sequencing libraries were generated using the Twist Human Core Exome Plus kit (Twist Bioscience). Libraries were sequenced on an Illumina platform by CeGaT (Tübingen, Germany). fastp[11] (v0.21.0) was used to remove artificial and low quality (Phred quality score below 15) sequences. Putative base calling errors located in regions where two reads of a read pair overlap were corrected (fastp option: '--correction'). The sequences were then aligned to the human reference assembly [NCBI GRCh38 (GCA_000001405.15)] with the Burrows-Wheeler Aligner (BWA mem, v0.7.17-r1188).[12] Strelka2 (v2.9.10)[13] and GATK4 (v4.1.9.0)[14] were used to detect genetic variation. Variants were annotated using the Ensembl Variant Effect Predictor (v103.0).[15]

The trio exome data were analyzed with respect to (i) *de novo* variants (allele frequency ≤0.5%), which are present in the proband and absent in both parents, (ii) autosomal recessive variants (allele frequency ≤0.5%), which are present in the compound heterozygous or homozygous state in the proband and heterozygous in the parents, (iii) X-linked variants (allele frequency ≤0.5%), which are present in the hemizygous state in the proband and inherited from the mother, and (iv) copy number variations (CNVs) of exonic and surrounding non-coding regions. Sequence variants are described according to the nomenclature of the Human Genome Variation Society (HGVS nomenclature). Variant prioritization was based on database queries and *in silico* pathogenicity programs (AlphaMissense,[16] CADD,[10] and REVEL[17]), splice site predictions (Alamut Visual Plus v1.4; SOPHIA GENETICS, providing: GeneSplicer,[5] MaxEntScan,[4] NNSplice 0.9,[3] and SpliceSiteFinder-like[2]), and exonic splicing enhancer (ESE) predictions (Alamut Visual Plus v1.4; SOPHIA GENETICS, providing: ESEfinder and RESCUE-ESE).[6,7]

Variant validation and segregation

Sanger sequencing permitted *NOP58* (NM_015934.5) variant validation and/or segregation in leukocyte-derived DNA from the proband, his healthy sister, and parents. PCR amplicons were generated according to standard PCR protocols with the OneTaq® Quick-Load 2 × Master Mix (New England Biolabs). Primers were designed to amplify the variant-containing exon 7 of *NOP58* (NM_015934.5) and adjacent intronic sequences (**Table S1**). Amplicons were directly sequenced using the ABI BigDye Terminator Sequencing Kit (Applied Biosystems) and an automated capillary sequencer (ABI 3500, Applied Biosystems). Sequence electropherograms were analyzed using SeqManPro™ (DNASTAR® Software for Life Scientists) and Chromas Lite 2.1.1 (Technelysium Pty Ltd). The *NOP58* variant was described according to the GenBank reference sequences NM_015934.5 and NP_057018.1. Correct variant nomenclature was assessed using Mutalyzer (https://mutalyzer.nl/name-checker).

Cell culture

Primary dermal fibroblasts were cultured from a skin biopsy of the proband and three healthy female controls (Ctrl. 1-3, all 4 years old) in Dulbecco's modified Eagle medium (DMEM; Thermo Fisher Scientific) supplemented with 10% fetal bovine serum (FBS; GE Healthcare) and penicillin-streptomycin (100 U/mL and 100 mg/mL, respectively; Thermo Fisher Scientific). The same passage number of proband and control fibroblasts was used in all experiments. Primary fibroblasts were regularly tested for mycoplasma contamination and confirmed to be mycoplasma-free.

RNA isolation and transcript analysis

300,000 primary fibroblasts from the proband and controls were seeded into 6 cm culture dishes. The following day, total RNA was extracted using the Monarch Total RNA Miniprep Kit (New England Biolabs). The RNA concentration and purity of the samples were assessed using the Epoch™ Microplate Spectrophotometer (Biotek Instruments). 1 µg of total RNA was reverse transcribed using the LunaScript®RT Super Mix Kit (New England Biolabs). Reverse transcription (RT)-PCR fragments were

generated according to standard PCR protocols with OneTaq® Quick-Load® 2× Master Mix (New England Biolabs). Primer sequences are described in **Table S1**. RT-PCR products were directly Sanger-sequenced.

Real-time quantitative PCR (RT-qPCR)

RT-qPCR was performed to determine relative *NOP58* mRNA, snoRNA, and rRNA levels. Technical triplicates of RT-qPCR samples were prepared as a 10-µL approach using the SYBR Green I- based Luna Universal qPCR Master Mix (New England Biolabs), 500 nM of each primer, and 1 µL of the reverse transcription reaction as described above. Primer sequences for RT-qPCR are described in **Table S1**. RT-qPCR was performed using the QuantStudio 3 Real-Time PCR System equipped with the QuantStudio Design&Analysis Software v1.4.3 (Thermo Fisher Scientific).

For relative *NOP58* mRNA and snoRNA analysis, the PCR conditions consisted of a pre-run at 95°C for 5 minutes, followed by 40 cycles of 30 seconds at 95°C, 30 seconds at 58°C, and 45 seconds at 72°C. The specificity of PCR amplification was determined by melting curve analysis with a range from 60°C to 95°C. The cycle threshold (CT) values of the target RNAs were normalized to the housekeeping mRNA of *GAPDH*. $2^{-\Delta Ct}$ values were calculated individually for each experiment and expressed as a fold change to the mean of the three controls. Canonically spliced *NOP58* mRNAs with exon 7 were targeted by using primers located in exons 6 and 7 and exons 7 and 8. Total *NOP58* mRNA levels were determined using primers located in exons 5 and 6 and exons 11 and 13. The proportion of *NOP58* mRNAs with exon 7 was calculated as the ratio of *NOP58* mRNA with exon 7 to total *NOP58* mRNA, using all respective primer combinations.

For rRNA analysis, the PCR conditions consisted of a pre-run at 95°C for 90 seconds, followed by 40 cycles of 10 seconds at 94°C, 30 seconds at 55°C, and 45 seconds at 72°C. The specificity of PCR amplification was determined by melting curve analysis with a range from 60°C to 95°C. The cycle threshold (CT) values of the target rRNA precursors were normalized to the total 18S rRNA amplified

using primers r18S-F and r18S-total-R (**Table S1**). $2^{-\Delta\Delta Ct}$ values were calculated individually for each experiment and expressed as a fold change to the mean of the three controls.

Antibodies and reagents

Primary antibodies and dilutions used: rabbit anti-NOP58 antibody (BethylLabs; #A302-719A; WB: 1:1,000 in 5% BSA/TBST), mouse anti-fibrillarin antibody (abcam; #ab18380; WB: 1:500 in 5% mTBST; IF: 1:100), mouse anti-NOP56 antibody (invitrogen; #MA5-24641; WB: 1:500 in 5% BSA/TBST), rabbit anti-SNU13/anti-NHP2L1 antibody (abcam; #ab95958; WB: 1:500 in 5% BSA/TBST), hFAB™ Rhodamine anti-GAPDH antibody (Bio-Rad; #12004167; WB: 1:10,000), and hFAB™ Rhodamine anti-tubulin antibody (Bio-Rad; #12004165; WB: 1:10,000).

Secondary antibodies and dilutions used: goat anti-mouse IgG:StarBright Blue 700 antibody (Bio-Rad; #12004159; WB: 1:7,500-10,000); goat anti-rabbit IgG:StarBright Blue 700 antibody (Bio-Rad; #12004162; WB: 1:7,500-10,000), and goat anti-mouse IgG secondary antibody Alexa Fluor 488-conjugated (Invitrogen; #A11029; IF: 1:1,000).

Reagent used: ProLong™ Diamond Antifade Mountant with DAPI (Invitrogen; #P36962).

Immunoblotting

150,000 fibroblasts were seeded into six-well plates. The following day, cells were harvested in ice-cold RIPA buffer [50 mM Tris-HCl, pH 8.0; 150 mM NaCl; 1% NP-40; 0.5% DOC (sodium deoxycholate); 0.1% SDS (sodium dodecyl sulfate)] supplemented with Mini Protease Inhibitor and PhosSTOP (Roche) and lysed on ice for 10 minutes. Cell debris was removed by centrifugation for 10 min at 4°C, 4x sample buffer (33% glycerol, 80 mM Tris-HCl pH 6.8, 0.3 M DTT, 6.7% SDS, and 0.1% bromophenol blue) was added to the supernatant, and samples were boiled at 95°C for 5 min. Equal amounts of whole-cell lysates were loaded and separated on a Mini PROTEAN® TGX Stain-Free Gel (Bio-Rad) under denaturing conditions, followed by transfer to polyvinylidene fluoride membranes. Membranes were blocked in 5% milk in TBST (mTBST) or 5% BSA in TBST and incubated with the primary antibody overnight in

blocking solution at 4°C. After washing, secondary antibodies were incubated for 1 hour in 0.5% mTBST or 0.5% BSA in TBST at room temperature. Immunoblots were digitally imaged using a ChemiDoc MP (Bio-Rad), with exposure time optimized to avoid saturation. Bands were automatically defined and intensities were determined using the built-in band detection tool of the Image Lab v6.0 software (Bio-Rad). Levels of target proteins were normalized to tubulin or GAPDH.

Immunofluorescence, confocal fluorescence microscopy, and quantitative analyses

30,000 fibroblasts of the proband and three controls were cultivated on glass coverslips in 12-well plates. The following day, cells were fixed with 4% paraformaldehyde (PFA) in phosphate-buffered saline (PBS) and washed three times with PBS. After treatment with permeabilization/blocking solution (2% bovine serum albumin; 3% goat serum; 0.5% Nonidet P40 in PBS), cells were incubated in antibody solution (3% goat serum; 0.1% Nonidet P40 in PBS) containing the primary antibody. Cells were washed with PBS and incubated with Alexa Fluor 488-conjugated secondary antibody. After extensive washing with PBS, cells were embedded in mounting solution with DAPI. Cells were analyzed with Leica TCS SP8 X confocal microscope (Microscope: Leica DMi8; Objective: 63× HC PL APO Oil CS2, NA: 1.4; Software: Leica LAS X SP8). ImageJ (v1.54f; NIH) was used for visualization.

Nucleolar structure was assessed on blinded samples after fibrillarin staining using two different criteria: 1) cells were categorized according to the number of condensates per cells, ranging from one to seven; 2) cells were categorized into two groups: (i) cells with normal nucleolar structure, showing at least one very bright spot within the condensates and (ii) cells with abnormal nucleolar structure, showing fluorescence signals evenly distributed throughout the condensates. Nucleolar structure was assessed in a minimum of 20 cells per fibroblast cell line and experiment.

Data analysis and statistics

Quantitative data are presented by Prism v10.2.3 software (GraphPad Software) as the mean ± standard deviation (SD). For quantification, one- or two-way ANOVA followed by a Dunnett's

post hoc test for multiple comparisons was performed. A $p \leq 0.05$ was considered statistically significant (*$p \leq 0.05$; **$p \leq 0.01$; ***$p \leq 0.001$; ****$p \leq 0.0001$).

SUPPLEMENTAL REFERENCES

1. Zhang, M.Q. (1998). Statistical features of human exons and their flanking regions. Hum Mol Genet *7*, 919-932. 10.1093/hmg/7.5.919.
2. Shapiro, M.B., and Senapathy, P. (1987). RNA splice junctions of different classes of eukaryotes: sequence statistics and functional implications in gene expression. Nucleic Acids Res *15*, 7155-7174. 10.1093/nar/15.17.7155.
3. Reese, M.G., Eeckman, F.H., Kulp, D., and Haussler, D. (1997). Improved splice site detection in Genie. J Comput Biol *4*, 311-323. 10.1089/cmb.1997.4.311.
4. Yeo, G., and Burge, C.B. (2004). Maximum entropy modeling of short sequence motifs with applications to RNA splicing signals. J Comput Biol *11*, 377-394. 10.1089/1066527041410418.
5. Pertea, M., Lin, X., and Salzberg, S.L. (2001). GeneSplicer: a new computational method for splice site prediction. Nucleic Acids Res *29*, 1185-1190. 10.1093/nar/29.5.1185.
6. Cartegni, L., Wang, J., Zhu, Z., Zhang, M.Q., and Krainer, A.R. (2003). ESEfinder: A web resource to identify exonic splicing enhancers. Nucleic Acids Res *31*, 3568-3571. 10.1093/nar/gkg616.
7. Fairbrother, W.G., Yeh, R.F., Sharp, P.A., and Burge, C.B. (2002). Predictive identification of exonic splicing enhancers in human genes. Science *297*, 1007-1013. 10.1126/science.1073774.
8. Chen, S., Francioli, L.C., Goodrich, J.K., Collins, R.L., Kanai, M., Wang, Q., Alfoldi, J., Watts, N.A., Vittal, C., Gauthier, L.D., et al. (2024). A genomic mutational constraint map using variation in 76,156 human genomes. Nature *625*, 92-100. 10.1038/s41586-023-06045-0.
9. Sun, K.Y., Bai, X., Chen, S., Bao, S., Zhang, C., Kapoor, M., Backman, J., Joseph, T., Maxwell, E., Mitra, G., et al. (2024). A deep catalogue of protein-coding variation in 983,578 individuals. Nature *631*, 583-592. 10.1038/s41586-024-07556-0.
10. Kircher, M., Witten, D.M., Jain, P., O'Roak, B.J., Cooper, G.M., and Shendure, J. (2014). A general framework for estimating the relative pathogenicity of human genetic variants. Nat Genet *46*, 310-315. 10.1038/ng.2892.
11. Chen, S., Zhou, Y., Chen, Y., and Gu, J. (2018). fastp: an ultra-fast all-in-one FASTQ preprocessor. Bioinformatics *34*, i884-i890. 10.1093/bioinformatics/bty560.
12. Li, H., and Durbin, R. (2010). Fast and accurate long-read alignment with Burrows-Wheeler transform. Bioinformatics *26*, 589-595. 10.1093/bioinformatics/btp698.
13. Kim, S., Scheffler, K., Halpern, A.L., Bekritsky, M.A., Noh, E., Kallberg, M., Chen, X., Kim, Y., Beyter, D., Krusche, P., and Saunders, C.T. (2018). Strelka2: fast and accurate calling of germline and somatic variants. Nat Methods *15*, 591-594. 10.1038/s41592-018-0051-x.
14. McKenna, A., Hanna, M., Banks, E., Sivachenko, A., Cibulskis, K., Kernytsky, A., Garimella, K., Altshuler, D., Gabriel, S., Daly, M., and DePristo, M.A. (2010). The Genome Analysis Toolkit: a MapReduce framework for analyzing next-generation DNA sequencing data. Genome Res *20*, 1297-1303. 10.1101/gr.107524.110.
15. McLaren, W., Gil, L., Hunt, S.E., Riat, H.S., Ritchie, G.R., Thormann, A., Flicek, P., and Cunningham, F. (2016). The Ensembl Variant Effect Predictor. Genome Biol *17*, 122. 10.1186/s13059-016-0974-4.
16. Cheng, J., Novati, G., Pan, J., Bycroft, C., Zemgulyte, A., Applebaum, T., Pritzel, A., Wong, L.H., Zielinski, M., Sargeant, T., et al. (2023). Accurate proteome-wide missense variant effect prediction with AlphaMissense. Science *381*, eadg7492. 10.1126/science.adg7492.
17. Ioannidis, N.M., Rothstein, J.H., Pejaver, V., Middha, S., McDonnell, S.K., Baheti, S., Musolf, A., Li, Q., Holzinger, E., Karyadi, D., et al. (2016). REVEL: An Ensemble Method for Predicting the Pathogenicity of Rare Missense Variants. Am J Hum Genet *99*, 877-885. 10.1016/j.ajhg.2016.08.016.
