# Supplementary material for: A homozygous synonymous NOP58 variant causes a neurodevelopmental disorder by impairing maturation of pre-ribosomal RNAs
Source: HGG Adv. 2025 Dec 11;7(1):100557. doi: 10.1016/j.xhgg.2025.100557 (PMC12800697; doi:10.1016/j.xhgg.2025.100557)
Supplement: Document S2. Article plus supplemental information [file mmc2.pdf]

HGG Advances

REPORT

A homozygous synonymous *NOP58* variant causes a neurodevelopmental disorder by impairing maturation of pre-ribosomal RNAs

Loisa D. Bonde,[1] Tess Holling,[1] Malik Alawi,[2] Ahmed A. El Beheiry,[3] Zabih Mir Hassani,[4] François Bachand,[4] Ibrahim M. Abdelrazek,[5] and Kerstin Kutsche[1,6,7,*]

Summary

Ribosomes are ribonucleoproteins that are responsible for protein synthesis. They consist of ribosomal proteins and ribosomal RNAs (rRNAs). Pre-rRNAs are co-transcriptionally processed and chemically modified. The 2′-*O*-methylation of rRNAs is guided by box C/D small nucleolar ribonucleoprotein particles (snoRNPs), which are composed of a box C/D snoRNA and the core proteins NOP56, NOP58, SNU13, and the methyltransferase fibrillarin. Catalytically active box C/D snoRNPs function in nucleoli. We performed trio whole-exome sequencing in a proband with a severe neurodevelopmental disorder including global developmental delay, microcephaly, seizures, and ophthalmological and brain abnormalities and his healthy parents and identified the homozygous synonymous variant c.516G>A; p.Leu172= in *NOP58*. In fibroblasts of the proband, we demonstrated skipping of exon 7 in most *NOP58* mRNAs, while ~20% canonically spliced *NOP58* transcripts were detected in the proband compared with control cells. NOP58 protein levels were reduced to ~12% in proband cells that concomitantly reduced fibrillarin levels. Analysis of nucleoli in proband-derived fibroblasts revealed changes in the number of nucleolar condensates and in nucleolar morphology. We found reduced levels of three box C/D snoRNAs required for 2′-*O*-methylation and of one box C/D snoRNA important for 2′-*O*-methylation and pre-rRNA processing. Analysis of pre-rRNA maturation by RT-qPCR revealed increased 45S and 21S pre-rRNA levels, whereas the amplification signal for the 47S, 32S, and 26S pre-rRNAs was substantially decreased in proband compared with control cells. Together, our data unveil that the homozygous *NOP58* variant c.516G>A represents a hypomorphic allele and underlies the neurodevelopmental phenotype in the proband, likely by impairing pre-rRNA maturation.

Ribosome biogenesis is an essential cellular process for embryonic development and cell survival.[1] The assembly of small and large ribosomal subunits that are the heart of mRNA translation requires the regulated action of over 250 ribosome biogenesis factors, including proteins and non-coding RNAs.[2,3] Ribosomal RNAs (rRNAs) are important for the catalytic activity of ribosomes. Pre-rRNA is transcribed by polymerase I and co-transcriptionally folded, chemically modified, and processed.[3] rRNA modifications, such as 2′-*O*-ribose methylation and pseudouridylation, serve to stabilize the ribosome core and are important for ribosome heterogeneity and the fine-tuning of ribosome function.[4,5] Small nucleolar RNAs (snoRNAs) are a highly abundant class of RNAs that function in the covalent modification and processing of pre-rRNAs in the nucleolus. Based on conserved sequence elements, snoRNAs are categorized into box H/ACA snoRNAs (*SNORA*s) and box C/D snoRNAs (*SNORD*s). For box H/ACA and box C/D snoRNAs, a specific set of core proteins associates with the snoRNA to form small nucleolar ribonucleoprotein particles (snoRNPs). The box H/ACA snoRNPs are required to isomerize uridine to pseudouridine in the rRNA, while the box C/D snoRNPs are responsible for transferring a methyl group to the 2′-hydroxyl of the ribose moiety of the rRNA. A subset of box H/ACA and C/D snoRNPs are required for pre-rRNA cleavage and folding events.[6–8]

The box C/D snoRNP forms by binding of a box C/D snoRNA to a set of core proteins including the methyltransferase fibrillarin, the related proteins NOP56 and NOP58, and SNU13.[9–11] For the assembly of box C/D snoRNPs, several proteins are required including the HSP90/R2TP complex, C12orf45, NUFIP, ZNHIT3, and ZNHIT6. The synthesis of box C/D snoRNAs and the maturation of the box C/D snoRNP occurs in the nucleus. Pre-snoRNP particles are then transported to Cajal bodies where the final remodeling and processing occurs. Catalytically active box C/D snoRNPs are targeted to nucleoli to function in ribose methylation of rRNA.[7] Base pairing between the snoRNA and the substrate RNA is required for fibrillarin-mediated 2′-*O*-methylation of rRNAs.[12,13]

Pathogenic variants in *ZNHIT3* (MIM: 604500) and *NOP56* (MIM: 614154) cause the progressive encephalopathy with edema, hypsarrhythmia, and optic atrophy

[1]Institute of Human Genetics, University Medical Center Hamburg-Eppendorf, Hamburg, Germany; [2]Bioinformatics Core, University Medical Center Hamburg-Eppendorf, Hamburg, Germany; [3]Radiodiagnosis and Intervention Radiology Department, Faculty of Medicine, Alexandria University, Alexandria, Egypt; [4]Department of Biochemistry and Functional Genomics, Université de Sherbrooke, Sherbrooke, QC, Canada; [5]Department of Human Genetics, Medical Research Institute, Alexandria University, Alexandria, Egypt; [6]German Center for Child and Adolescent Health (DZKJ), Partner Site Hamburg, Hamburg, Germany
[7]Lead contact
*Correspondence: kkutsche@uke.de
https://doi.org/10.1016/j.xhgg.2025.100557.
© 2025 The Author(s). Published by Elsevier Inc. on behalf of American Society of Human Genetics.
This is an open access article under the CC BY license (http://creativecommons.org/licenses/by/4.0/).

(PEHO) syndrome (MIM: 260565) and spinocerebellar ataxia 36 (SCA36 [MIM: 614153]), respectively.[14,15] A heterozygous GGCCTG repeat expansion in intron 1 of *NOP56* underlies SCA36.[15] Bi-allelic *ZNHIT3* missense variants have been reported in subjects with PEHO syndrome, while a missense in *trans* with a frameshift variant has recently been identified in two fetuses with hydrops from one family, resulting in pregnancy loss.[14,16,17] Functional studies in human cell lines and yeast have shown that *ZNHIT3* pathogenic variants reduce steady-state levels of ZNHIT3 proteins and of box C/D snoRNAs, impair rRNA processing or decrease rRNA levels, change rRNA modification, and reduce cellular translation.[14,17,18]

For this study, the proband's parents provided written informed consent for participation, clinical data and specimen collection, genetic analysis, and publication of relevant findings, including facial photographs and brain MRI scans, under a protocol approved by the Ethics Committee of the Hamburg Medical Chamber (PV7038-4438-BO-ff; Hamburg, Germany). Detailed methodologies are given in the supplemental information.

A 28-month-old male was born full-term as the second child of healthy consanguineous parents via Cesarean section following an uncomplicated pregnancy. His birth weight was 3.5 kg. Shortly after birth, he was admitted to the NICU for 7 days due to neonatal jaundice. He had a healthy younger sister and an older brother with a similar condition, who exhibited global developmental delay and severe microcephaly, with prenatal ultrasound showing alobar holoprosencephaly (Figure 1A). His brother passed away at the age of 7 months due to a respiratory infection. The proband developed focal to bilateral seizures at the age of 4 months.

By the age of 28 months, the proband had global developmental delay, characterized by an inability to sit independently, limited vocalizations, and poor visual attention. At examination, he had severe microcephaly, brachycephaly, receding anterior hairline, prominent metopic ridge, thin eyebrows, hypotelorism, narrow and upslanted palpebral fissures, epicanthus, strabismus, blue sclera, wide nasal ridge, long and deep philtrum, thin upper lip vermilion, full cheeks, and prominent antihelix (Figure 1B). His growth parameters at the age of 28 months were as follows: an occipitofrontal head circumference of 37.5 cm (−6.39 z), along with a decreased length of 81.5 cm (−2.25 z) and weight of 11.5 kg (−0.92 z).

Brain imaging at age 18 months revealed a severe microcephalic configuration of the skull, accompanied by a marked reduction in the entire brain volume, particularly in the supratentorial region. The imaging also showed a simplified gyral pattern, and hypoplasia of the corpus callosum, optic nerve, and optic chiasm (Figure 1C). His fundoscopic examination identified bilateral microphthalmia, microcornea, optic disc pallor, and optic atrophy. Echocardiography, hearing assessment, and abdominal and pelvic ultrasound were unremarkable. His karyotype was normal (46,XY).

We performed trio whole-exome sequencing in the proband and healthy parents and did not detect any rare, likely pathogenic variant in a known disease gene. In the proband, we identified a homozygous synonymous variant in exon 7 of the candidate gene *NOP58* (MIM: 616742), GenBank: NM_015934.5:c.516G>A; p.Leu172= (Figure 2A), which was confirmed by Sanger sequencing in the proband in the homozygous state and in his healthy sister and both parents in the heterozygous state (Figure 1D; Table S1). Material from the similarly affected older brother was not available for segregation analysis. The *NOP58* variant is absent in the gnomAD database (v.4.1.0)[19] and in the Regeneron Genetics Center Million Exome data (Table S2).[20] With a loss-of-function observed/expected upper bound fraction of 0.995 and a *Z* score of 0.93, *NOP58* is not intolerant to loss-of-function and missense variants, respectively (Database: gnomAD v.4.1.0).[19] Splice site prediction programs did not predict any change for the detection of the canonical splice acceptor in intron 6 of *NOP58* (Figure S1A; Table S2). Creation of a new splice site in exon 7 due to the G-to-A change was also not predicted. However, when we looked at exonic splicing enhancers using ESEFinder,[21] we found that the binding site for the two splicing factors SRSF2 and SRSF5 was lost in exon 7 due to the c.516G>A change (Figure S1). We received a single match from GeneMatcher[22] that was an individual with intellectual disability, autism, status epilepticus, hippocampal atrophy, and a *de novo* missense variant in *NOP58*. As there was only partial overlap between the phenotype of our proband and that of the subject from GeneMatcher, and the zygosity of the variants differed, we decided to investigate the functional consequences of the homozygous synonymous *NOP58* variant. We obtained primary skin fibroblasts from the proband and performed *NOP58* transcript analysis and various biochemical and cell biology experiments that collectively demonstrate clinical relevance of the bi-allelic *NOP58* variant.

We investigated the effect of the *NOP58* variant c.516G>A in exon 7 on *NOP58* pre-mRNA splicing using RNA (cDNA) isolated from fibroblasts of the proband and controls. We used a forward primer in exon 5 (c5F) and a reverse primer in exon 8 (c8R) in RT-PCR experiments (Figure 2A; Table S1). In control 1 cells, we observed a strong RT-PCR band of the expected wild-type size (275 bp) in the agarose gel, while there was only a very faint 275-bp band in proband cells. In addition, a prominent smaller band of ∼140 bp was observed in proband cells (Figure 2B). Sequencing of the 275-bp RT-PCR product from control 1 revealed the reference sequence, while direct sequencing of the smaller RT-PCR amplicon from the proband identified aberrantly spliced *NOP58* transcripts with exon 6 directly spliced to exon 8 (Figure 2D). Skipping of exon 7 causes loss of 135 nucleotides in *NOP58* transcripts and is predicted to lead to loss of 46 amino acid residues and insertion of a cysteine at the protein

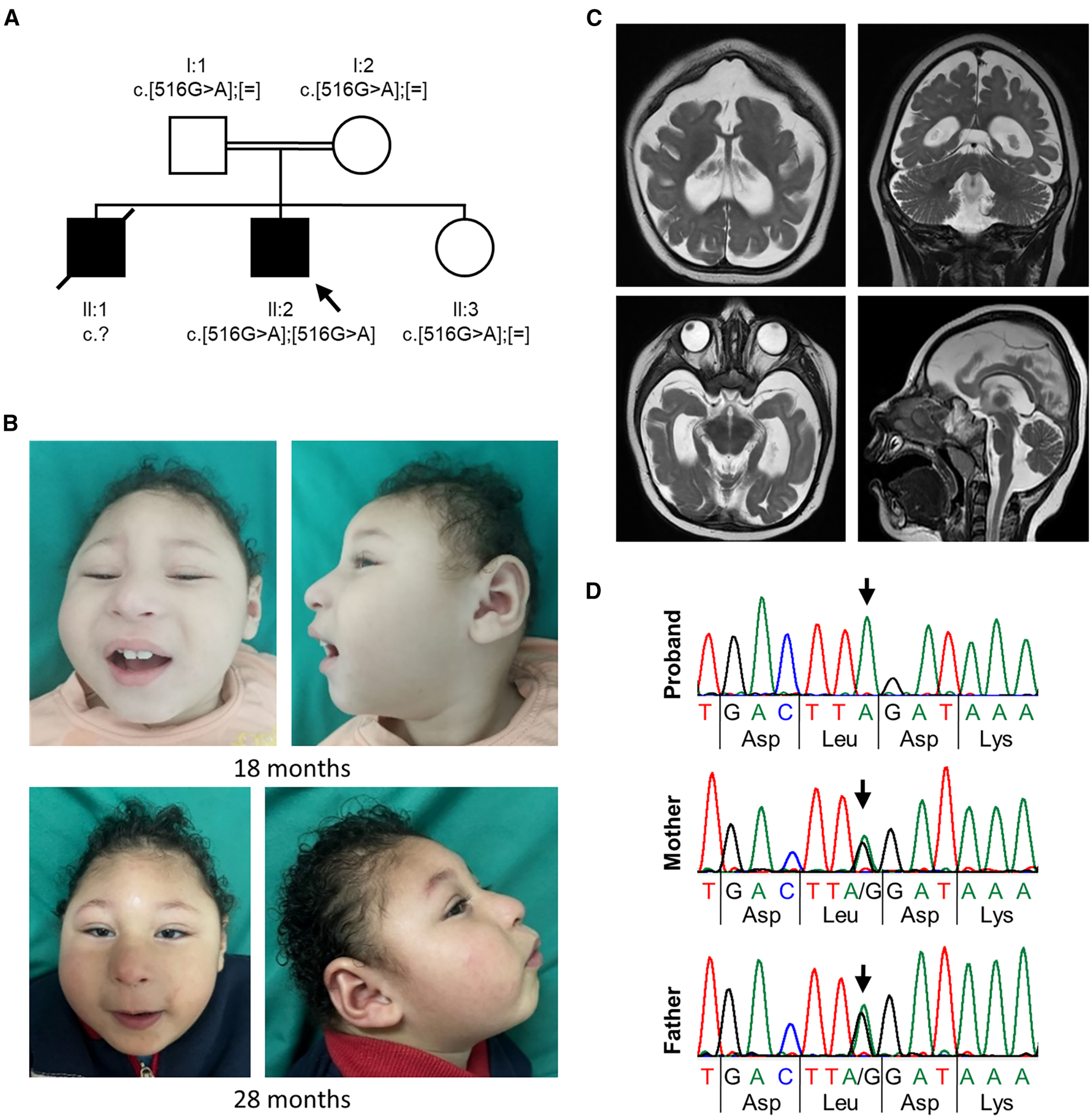

Figure 1. Pedigree, variant segregation, photographs, and brain MRI scans of the proband with the homozygous synonymous *NOP58* c.516G>A variant

(A) Pedigree of the family. The healthy father (I:1) and the healthy mother (I:2) are first-degree cousins and heterozygous carriers of the NM_015934.5:*NOP58* c.516G>A; p.Leu172= variant. The proband (II:2, marked with an arrow) carries the *NOP58* c.516G>A variant in the homozygous state. His similarly affected older brother (II:1) died at the age of 7 months, but could not be genetically tested due to lack of material. The proband has one healthy sister (II:3) who carries the *NOP58* c.516G>A variant in the heterozygous state.

(B) Facial photographs of the proband at the age of 18 months (top) and 28 months (bottom) show brachycephaly, prominent metopic ridge, receding anterior hairline, prominent antihelix, thin eyebrows, hypotelorism, narrow and upslanted palpebral fissures, epicanthus, strabismus, microphthalmia, microcornea, blue sclera, wide nasal ridge, long and deep philtrum, thin upper lip vermilion, and full cheeks.

(C) Brain MRI scans of the proband at age 18 months. Axial and coronal T2-weighted images (top) show significant reduction in volume of the supratentorial brain with evidence of simplified gyral pattern, dilated bodies of the lateral ventricles tapering anteriorly, and slanted frontal convexity, more evident in the axial view. Axial T2-weighted image at lower level (bottom left) shows dilated temporal horns of the lateral ventricles along with thinning of the cisternal prechiasmatic segments of the optic nerves. Sagittal T2-weighted image (bottom right) shows a hypoplastic corpus callosum.

(D) Partial sequence electropherograms showing the *NOP58* c.516G>A variant in the homozygous state in the proband, and in the heterozygous state in the healthy parents (mother and father). Arrows point to the G-to-A change.

level (NM_015934.5:r.500_634del; p.Ser167_Gly212delinsCys). To analyze whether proband cells still express *NOP58* transcripts with exon 7, we performed RT-PCR using a forward primer in exon 5 (c5F) and a reverse primer in exon 7 (c7R) (Figure 2A). We obtained the expected RT-PCR product of 189 bp in control 1 and 2 and proband cells (Figure 2C). Sequencing of the amplicon identified canonically spliced *NOP58* transcripts with the

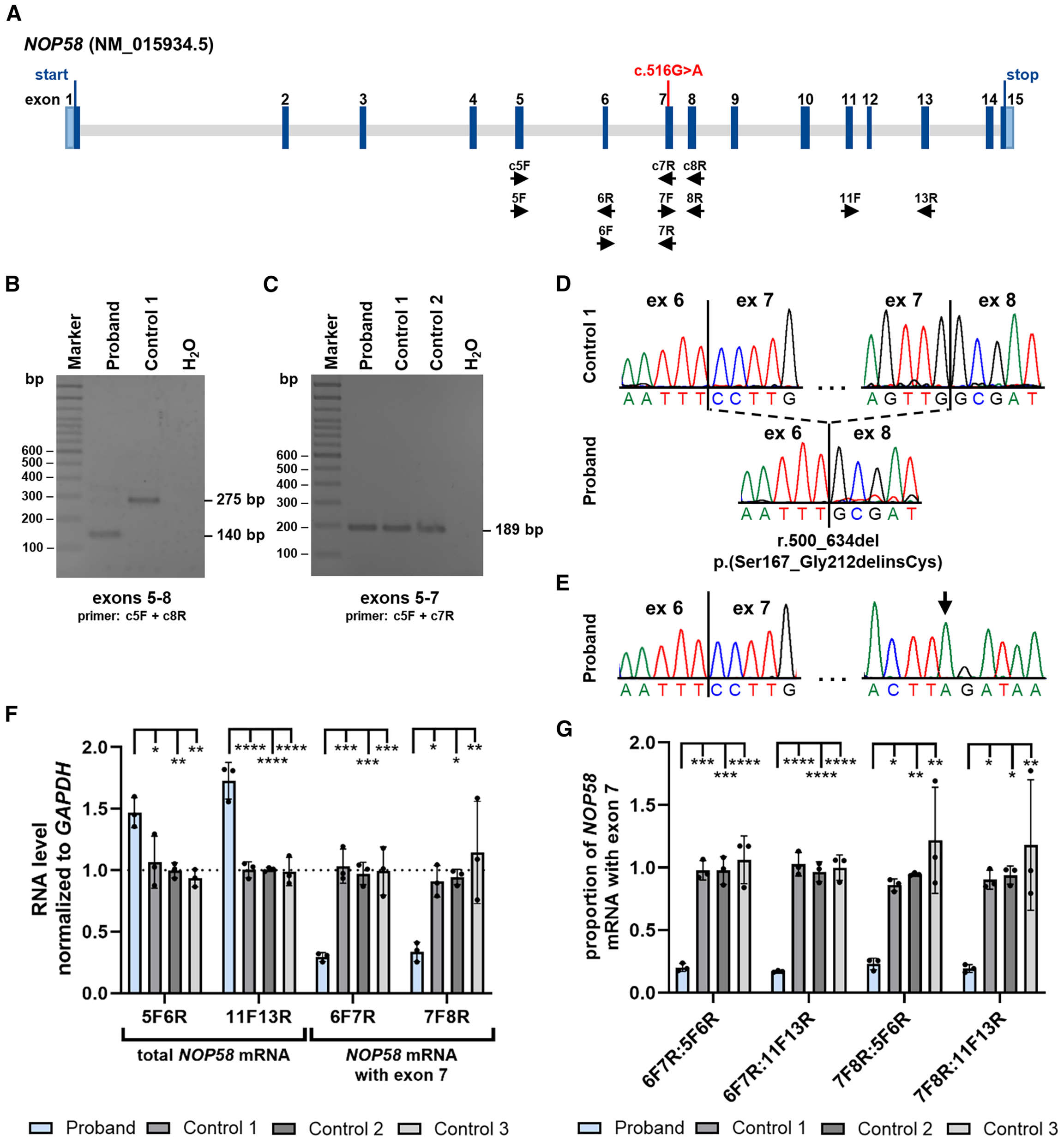

Figure 2. The homozygous *NOP58* c.516G>A variant leads to aberrant splicing of *NOP58* pre-mRNA

(A) Exon-intron structure of the *NOP58* gene based on the mRNA reference sequence NM_015934.5, showing the c.516G>A variant located in exon 7. Exons are represented as boxes and introns as gray lines. Untranslated regions are depicted in light blue, while the coding region is shown in dark blue. Start and stop codons are indicated. The primers used for qualitative and quantitative RT-PCR experiments are shown below the exon-intron structure.

(B and C) Agarose gels (2%) showing RT-PCR amplicons from fibroblast-derived cDNA of proband and control fibroblasts. (B) Using primers located in exons 5 (c5F) and 8 (c8R), the expected RT-PCR product of 275 bp was amplified in control 1. In contrast, a major amplicon of ~140 bp was obtained from cDNA of proband-derived cells. (C) Using primers located in exons 5 (c5F) and 7 (c7R), the expected RT-PCR product of 189 bp was amplified from cDNA of control 1 and 2 and proband cells.

(D) Direct sequencing of the RT-PCR amplicons obtained with primers located in exons 5 and 8 shown in (B). Partial sequence electropherograms show the canonically spliced *NOP58* transcript with exon 6 spliced to exons 7 and 8 in control 1 (top) and aberrantly spliced *NOP58* transcripts in the proband (bottom). Skipping of exon 7 (r.500_634del) is predicted to lead to an *in-frame* loss of 46 amino acid residues and insertion of a cysteine (p.Ser167_Gly212delinsCys) at the protein level.

(E) Direct sequencing of the RT-PCR amplicons obtained with primers located in exons 5 and 7 shown in (C). The partial sequence electropherograms of the proband show canonically spliced *NOP58* transcripts containing exon 7. The arrow points to the r.516G>A variant in exon 7.

(F and G) Relative quantification of *NOP58* mRNA levels by RT-qPCR using fibroblast-derived cDNA from cells of the proband and controls 1–3. A total of 300,000 primary fibroblasts from the proband and controls was seeded. The bars and errors show the mean ± SD of three independent experiments, each performed in triplicate. Individual data points are shown. One-way ANOVA followed by Dunnett's post hoc test was used for statistical analysis. (F) For quantification of total *NOP58* mRNA, primers located in exons 5 and 6 (F5R6) or exons 11 and 13 (11F13R) were used (left panel). To amplify only canonically spliced *NOP58* mRNAs with exon 7, primers located in

(legend continued on next page)

r.516G>A change in proband cells (Figure 2E). The data suggest that the homozygous *NOP58* c.516G>A variant causes preferential skipping of exon 7 in *NOP58* pre-mRNAs, while leaving some transcripts intact.

Next, we studied total *NOP58* mRNA levels by RT-qPCR using two different primer combinations that are located outside the aberrantly spliced region. With both primer pairs (5F and 6R and 11F and 13R; Figure 2A), we detected total *NOP58* mRNA levels that were 1.5- to 1.7-fold increased in proband compared with control cells (Figure 2F, left panel). To determine levels of canonically spliced *NOP58* mRNAs in proband cells, we used two primer combinations, one with a forward primer in exon 6 (6F) and a reverse primer in exon 7 (7R) and another with a forward primer in exon 7 (7F) and a reverse primer in exon 8 (8R) (Figure 2A). For both primer combinations, we found that levels of *NOP58* mRNAs with exon 7 were significantly decreased by ~3.3-fold in proband compared with control cells (Figure 2F, right panel). We next wanted to determine the proportion of canonically spliced *NOP58* mRNAs in proband cells. For this, we calculated the ratio of *NOP58* mRNA levels with exon 7 to total *NOP58* mRNA levels in proband and control cells, using values obtained from RT-qPCR experiments with four different primer pairs (see Figure 2F). As shown in Figure 2G, the proportion of *NOP58* mRNAs with exon 7 was between 86% and 122% in control cells versus 17% and 23% in proband cells. Together, these data show that total *NOP58* mRNA levels were significantly increased in proband cells, likely due to enhanced transcription compensating for potential NOP58 deficiency. Nonetheless, the proportion of correctly spliced *NOP58* transcripts was drastically reduced in fibroblasts of the proband, potentially leading to the production of a small amount of NOP58 wild-type protein.

Studies in human cell lines and yeast showed that *ZNHIT3* missense variants compromise the protein stability of ZNHIT3 and reduce the steady-state levels of ZNHIT3's binding partner NUFIP1.[14,17,18] We therefore assessed if the homozygous *NOP58* variant impacts steady-state levels of NOP58 and of all other components of the C/D box snoRNP complex in proband and control cells. Fibroblasts of the proband showed statistically significant reductions in the amount of NOP58 and fibrillarin, to ~12% and ~30%, respectively, compared with control cells (Figures 3A and 3B). However, levels of NOP56 and SNU13 were similar in proband and control cells (Figures 3A and 3B). The data show that fibroblasts of the proband have a residual amount of NOP58, which likely represents wild-type protein produced from the canonically spliced *NOP58* mRNAs. While the drastically decreased NOP58 amount concomitantly reduces steady-state levels of the methyltransferase fibrillarin in proband cells, levels of NOP56 and SNU13 do not seem to be affected.

The function of the box C/D snoRNP complex is important for efficient assembly of ribosomes in the nucleolus, a multilayered biomolecular condensate.[7,23] The nucleolar localization of the box C/D snoRNP requires all four core box C/D proteins.[24] We therefore analyzed the nucleolar morphology of proband and control cells by staining of fibrillarin, a nucleoli marker, followed by immunofluorescence analysis and confocal microscopy. As shown in Figures 4A and S2, the distribution of the fibrillarin signal was different in cells of the proband compared with control cells, with nucleoli showing a textured structure and bright spots in the condensates of control cells, whereas a more uniform signal distribution was found in nucleoli of proband cells. We first counted the number of nucleolar condensates per cell and identified a statistically significantly higher proportion of cells with a single condensate in proband (~38%) compared with control cells (9%–13%) (Figure 4B). In contrast, the percentage of cells with two and more condensates was similar in proband and control cells (Figure 4B). We next analyzed the nucleolar structure and categorized the cells as follows: (1) cells with a normal nucleolar structure, showing at least one very bright spot within the condensates and (2) cells with an abnormal nucleolar structure, showing evenly distributed fibrillarin signals throughout the condensates. We identified ~57% of proband fibroblasts with an abnormal nucleolar structure compared with 10%–12% in control cells (Figure 4C). Together, the data indicate significant changes in nucleolar condensates and morphology in fibroblasts of the proband.

Depletion of Nop58p, the NOP58 ortholog in yeast, causes a reduction of all five tested box C/D snoRNAs, while snoRNAs of the box H/ACA snoRNP complex were not affected.[9] Similarly, ectopic expression of disease-associated ZNHIT3 variant proteins in HEK293T cells decreased the steady-state levels of some methylating box C/D snoRNAs, while levels of box C/D snoRNAs involved in rRNA processing were not changed.[17] We next analyzed levels of six box C/D snoRNAs in proband and control fibroblasts. This included *SNORD91B*, *SNORD93*, and *SNORD125* that are important for 2′-*O*-methylation of rRNAs and *SNORD3A* and *SNORD18A* required for pre-rRNA processing.[17,25,26] The box C/D snoRNA *SNORD14A* has a dual role in production and 2′-*O*-methylation of rRNAs.[27,28] Levels of *SNORD14A*, *SNORD91B*, *SNORD93*, and *SNORD125* were significantly decreased to ~57%, ~55%, ~47%, and ~63%,

exons 6 and 7 (6F7R) or exons 7 and 8 (7F8R) were used (right panel). The amount of *NOP58* mRNA relative to *GAPDH* mRNA is presented. (G) The proportion of canonically spliced *NOP58* mRNAs with exon 7 is shown by calculating the ratio of *NOP58* mRNA with exon 7 to total *NOP58* mRNA. Ratios were calculated from relative *NOP58* mRNA levels using the primer combinations shown in (F) and as indicated below the graph. *$p \leq 0.05$, **$p \leq 0.01$, ***$p \leq 0.001$, ****$p \leq 0.0001$. bp, base pairs; ex, exon; F, forward primer; R, reverse primer.

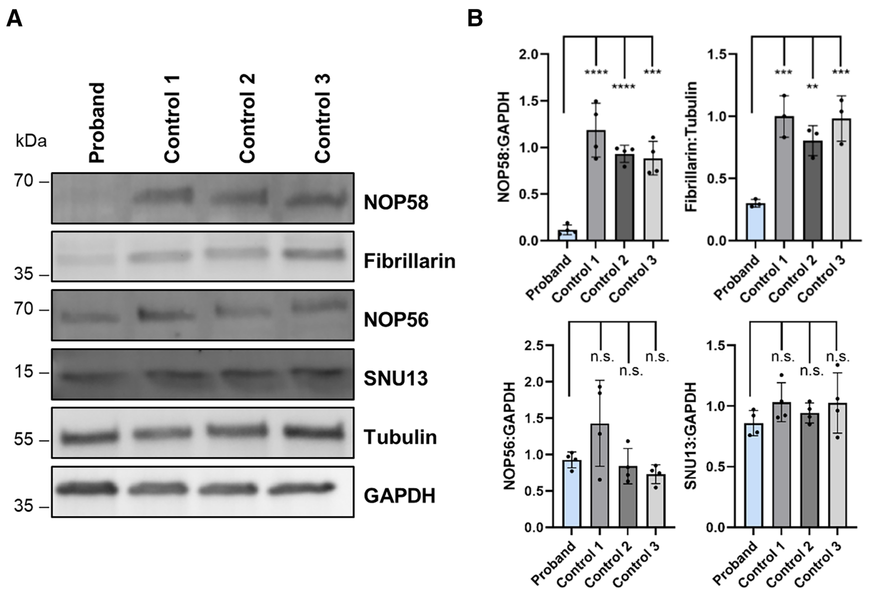

Figure 3. Levels of some components of the box C/D snoRNP complex are decreased in fibroblasts of the proband

(A) Representative immunoblots of whole-cell lysates from proband and control fibroblasts. A total of 150,000 fibroblasts from the proband and controls was seeded. Equal amounts of whole-cell lysates were loaded. Endogenous NOP58, fibrillarin, NOP56, and SNU13 were monitored with the indicated antibodies. Anti-tubulin and anti-GAPDH antibodies were used to control for equal loading.

(B) Quantification of protein levels from immunoblots shown in (A). Band intensities of fluorescence signals were quantified using the ChemiDoc imaging system. Levels of target proteins were normalized to tubulin or GAPDH. The bars and errors show the mean ± SD of three or four independent experiments. One-way ANOVA followed by Dunnett's post hoc test was used for statistical analysis. **$p \leq 0.01$, ***$p \leq 0.001$, ****$p \leq 0.0001$. kDa, kilodalton; n.s., not significant.

respectively, in proband compared with all control fibroblasts (Figure 5A). For *SNORD18A*, we found a decrease to ~72% in cells of the proband compared with controls that was only statistically significant compared with control 1 cells (Figure 5A). Levels of *SNORD3A* were similar in proband and control cells (Figure 5A). We also analyzed levels of *SNORA30*, a snoRNA of the box H/ACA snoRNP complex,[30] and identified a decrease in the levels of this snoRNA to ~76% in the proband that was only statistically significant when compared with control 1 cells (Figure 5A). Our data show that the steady-state levels of all tested box C/D snoRNAs guiding 2′-*O*-methylation and of *SNORD14A*, which is involved in both production and 2′-*O*-methylation of rRNAs, were consistently reduced in proband cells.

Reduced levels of certain box C/D snoRNAs, particularly *SNORD14A*, may suggest that pre-rRNA processing is altered in proband cells. To explore this possibility, we performed RT-qPCR using nine primer pairs that specifically target rRNA precursors and thereby monitor pre-rRNA processing in proband and control fibroblasts (Figure 5B). Similar levels of the 47S (primer pair spanning cleavage site 02), 30S, 21SC, and 18SE pre-rRNAs were observed in proband and control cells (Figure 5C). In contrast, the 45S and 21S rRNA precursors showed significantly increased levels in the proband cells (Figure 5C). Notably, the amplification signal for the 47S (primer pair spanning cleavage site 01), 32S, and 26S pre-rRNAs was substantially decreased in the proband compared with control cells (Figure 5C). This result was particularly unexpected, as the two primer pairs targeting different cleavage sites (01 and 02) of the same 47S pre-rRNA yielded contradictory results. Together, the increased levels of the 45S and 21S rRNA precursors in the proband's fibroblasts support an impairment in pre-rRNA processing; yet, the near-complete failure to amplify an RT-PCR product with three primer pairs remains to be elucidated.

The 28-month-old male proband with the homozygous synonymous variant c.516G>A; p.Leu172= in *NOP58* had a severe neurodevelopmental disorder, including severe global developmental delay, microcephaly, epilepsy, facial dysmorphism, microphthalmia, and other ophthalmological abnormalities. The proband had abnormal cerebral morphology, such as reduced brain volume, simplified gyral pattern, and hypoplastic corpus callosum. Our functional studies using proband-derived fibroblasts discovered skipping of exon 7 in most of the *NOP58* mRNAs. However, ~20% canonically spliced *NOP58* mRNAs were identified in proband cells that likely account for the production of a small amount of normal NOP58 protein in the fibroblasts. The residual amount of NOP58 is likely needed for the biogenesis and function of the box C/D snoRNP and may be compatible with life, suggesting that the *NOP58* variant c.516G>A; p.Leu172= is a hypomorphic rather than a complete loss-of-function allele. In line with this, deletion of the NOP58 ortholog in yeast (Nop58p) causes lethality, while its depletion impairs growth.[9,31] The three known PEHO syndrome-associated *ZNHIT3* missense variants p.Cys14Arg, p.Cys14Phe, and p.Ser31Leu allow embryonic development by destabilizing the ZNHIT3 protein leading to decreased ZNHIT3 steady-state levels in yeast and human cell culture.[14,17,18] In contrast, a *ZNHIT3* loss-of-function allele, such as the frameshift variant c.251_254del; p.Glu84Alafs*8, in *trans* with the p.Cys14Arg variant cause hydrops fetalis followed by early pregnancy loss.[17] Together, the data suggest that some residual amount of (less) functional box C/D snoRNPs is required for embryonic and cellular survival. Nonetheless, bi-allelic

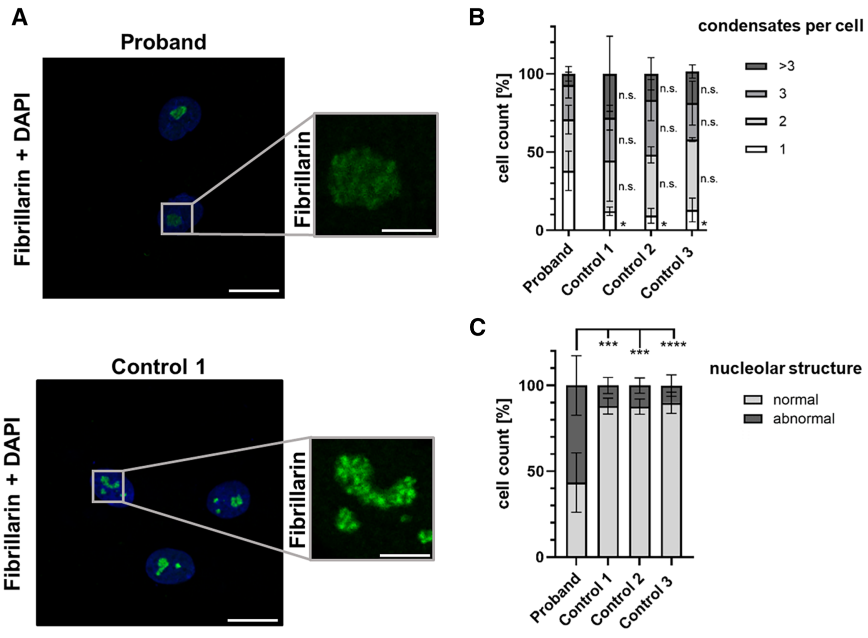

Figure 4. Nucleolar morphology is altered in proband-derived fibroblasts
(A) Immunofluorescence analysis using an anti-fibrillarin antibody followed by anti-mouse Alexa Fluor 488-conjugated secondary antibody (green) to stain nucleoli in proband and control fibroblasts. Nuclear DNA was stained with DAPI (blue). Fibroblasts were seeded on coverslips, cultivated under basal conditions, and imaged by confocal fluorescence microscopy. Representative image of proband (top) and control cells (bottom) is shown. Scale bars, 10 μm (left) and 5 μm (right).
(B and C) Quantification of nucleolar features in fibroblasts of the proband and three controls. The bars and errors show the mean ± SD of three independent experiments. Samples were blinded for analyses. A minimum of 20 cells per cell line was analyzed in each experiment. Two-way ANOVA followed by Dunnett's post hoc test was used for statistical analysis.
(B) Cells were classified based on the number of nucleolar condensates per cell, ranging from one to seven. Cells with more than three condensates were grouped together.
(C) Cells were categorized into two groups: (1) cells with normal nucleolar structure (i.e., cells with a textured fluorescence signal, showing at least one very bright dot within the condensate) and (2) cells with abnormal nucleolar structure (i.e., cells with evenly distributed fluorescence signals throughout the condensate). *$p \leq 0.05$, ***$p \leq 0.001$, ****$p \leq 0.0001$; n.s., not significant.

pathogenic variants in *ZNHIT3* and *NOP58* likely affecting the biogenesis of box C/D snoRNPs have severe consequences for neuronal and brain development in humans.

In fibroblasts of the proband with the homozygous *NOP58* variant, levels of the two core box C/D snoRNP proteins SNU13 and NOP56 were similar to control cells, while those of the methyltransferase fibrillarin were significantly reduced. This is in contrast to normal fibrillarin (Nop1p) levels in a yeast strain depleted of Nop58p.[9] The ZNHIT3-Cys14Phe variant protein concomitantly reduces endogenous NUFIP1 levels in HEK293T cells; however, steady-state levels of SNU13 and NOP58 are not affected.[17] In addition, ZNHIT3-Cys14Phe and -Ser31Leu variant proteins form complexes with NUFIP1 similar to wild-type ZNHIT3.[14,17] The data provide further evidence for an impaired, but not abolished, snoRNP biogenesis due to hypomorphic variants in *ZNHIT3* and *NOP58*. Furthermore, the data suggest that different human cells, as well as yeast, have distinct compensatory mechanisms to respond to the depletion of key box C/D snoRNP components or assembly factors.

Our data show that the steady-state levels of some box C/D snoRNAs are significantly reduced in proband-derived fibroblasts with the homozygous *NOP58* variant, whereas levels of other snoRNAs are only slightly affected, if at all. A consistent reduction was found for the three analyzed methylating box C/D snoRNAs *SNORD91B*, *SNORD93*, and *SNORD125*, while variable or no reduction was detected for the pre-rRNA processing box C/D snoRNAs and a box H/ACA snoRNA. Interestingly, the steady-state levels of the same three snoRNAs *SNORD91B*, *SNORD93*, and *SNORD125* are also reduced in HEK293T cells expressing PEHO-associated ZNHIT3 variant proteins. Other box C/D snoRNAs involved in methylation and pre-rRNA processing, as well as orphan box C/D snoRNAs, are not changed in this cellular system.[17] In yeast, Nop58p depletion and the introduction of the PEHO syndrome-causing *ZNHIT3* variants C11F and S29L (corresponding to p.Cys14Phe and p.Ser31Leu in human) result in lower levels of both rRNA modifying and processing box C/D snoRNAs.[9,18] The data suggest that some snoRNAs are more sensitive to the depletion of an important assembly or core protein of the box C/D snoRNP than others.

The consequences of the pathogenic *ZNHIT3* missense variants on rRNA modification, rRNA processing, and cellular translation have been investigated in various cellular and model systems. In yeast mutants and lung tissue of an affected fetus, rRNA hypomethylation of specific 2′-*O*-methylation sites was detected, rather than a general decrease in 2′-*O*-methylation of rRNAs. The defect in box C/D snoRNP assembly caused by *ZNHIT3* pathogenic variants results in a reduction of mature rRNA levels in a human cell line, rRNA processing impairments in yeast, and a decrease in global translation in both yeast and human cell culture.[17,18] Nop58p depletion in yeast causes severe pre-rRNA processing defects.[9] A first hint that rRNA homeostasis and/or the translational program may be impaired in proband-derived fibroblasts with the homozygous *NOP58* variant is that the number and morphology of nucleolar condensates are altered compared with control fibroblasts. The nucleolus serves an important biological role as a site of ribonucleoprotein particle and early

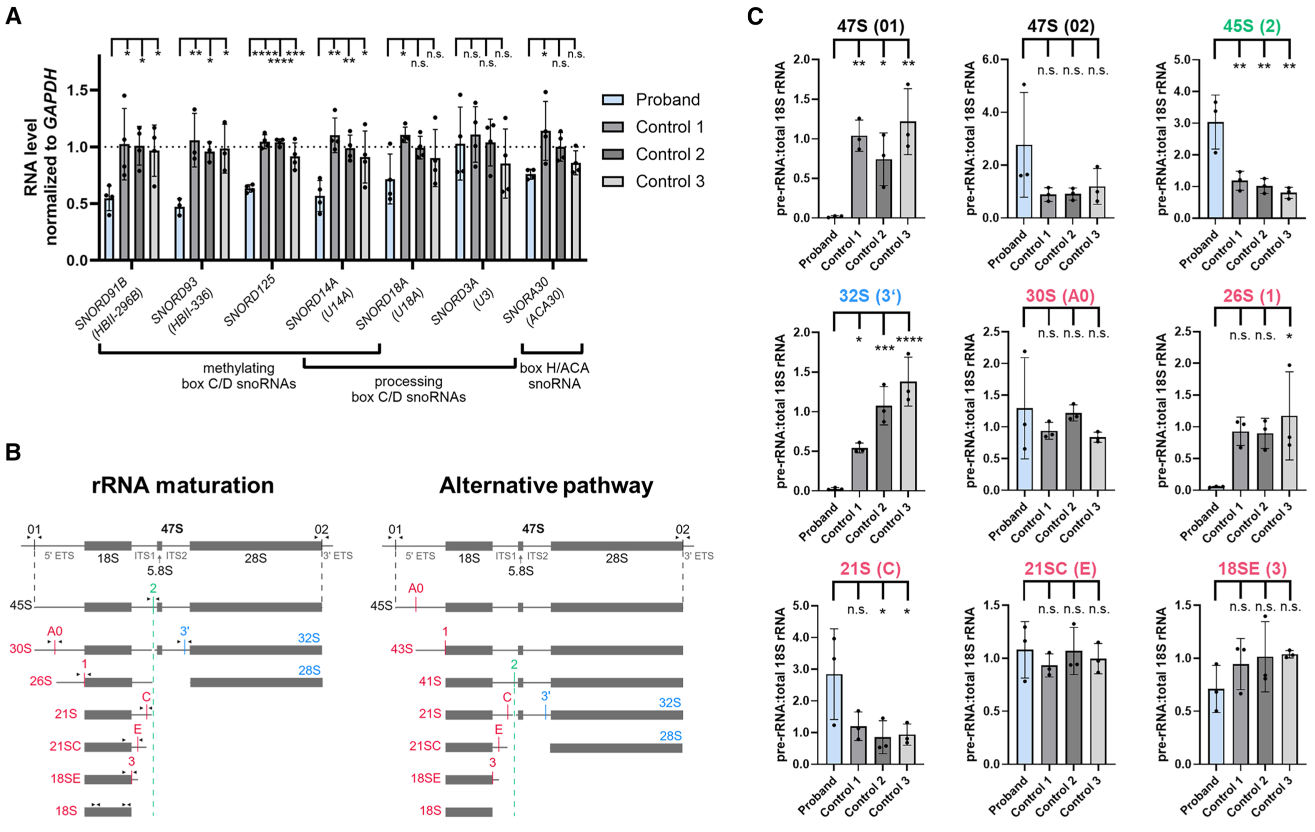

Figure 5. Reduced levels of certain snoRNAs and impaired pre-rRNA processing in fibroblasts of the proband
(A) Quantification of steady-state snoRNA levels in proband and control fibroblasts. A total of 300,000 primary fibroblasts from the proband and controls was seeded. For quantification, RNA levels were normalized to *GAPDH* levels. The bars and errors show the mean ± SD of three or four independent experiments, each performed in triplicate. Individual data points are shown. One-way ANOVA followed by Dunnett's post hoc test was used for statistical analysis. The gene symbol and the canonical name (shown in brackets below the gene symbol) of the analyzed snoRNAs are displayed beneath the graph. The type and function of the snoRNAs are also indicated.
(B) Pre-rRNA processing in human cells.[29] The earliest species of rRNA is the 47S pre-rRNA, which is cleaved at sites 01 and 02 (in black) to generate the 45S precursor. At this point, the maturation process can take place in one of the two possible pathways. In one pathway (rRNA maturation, left panel), 45S pre-rRNA processing continues by cleavage at site 2 (in green) within ITS1, generating the 30S and 32S species. Once cleavage at site 2 takes place, the resulting 30S species is further trimmed at A0 and 1 (in red), generating the 21S rRNA. In an alternative rRNA maturation pathway (right panel), concomitant cleavage at site A0 and 1 in the 45S precursor may precede cleavage at site 2. This can result in the generation of 43S and 41S species. The 21S is then further trimmed at sites C and E (in red), generating the 18SE, which is exported to the cytoplasm where it is cleaved at site 3 (in red), generating the mature 18S rRNA. In parallel, cleavage at site 3' (blue) is required for the production of mature 28S rRNA.
(C) Relative quantification of pre-rRNA levels by RT-qPCR using fibroblast-derived cDNA from cells of the proband and controls 1–3. For each primer pair, the forward and reverse primers are positioned directly upstream and downstream of the respective cleavage site shown in (B) (arrowheads) and were designed to specifically amplify the corresponding rRNA precursor indicated above each panel (nomenclature refers to the left panel in (B) [rRNA maturation pathway]). The corresponding cleavage site is given in parentheses. For quantification, pre-rRNA levels were normalized to total 18S rRNA levels. The bars and errors show the mean ± SD of three independent experiments, each performed in triplicate. Individual data points are shown. One-way ANOVA followed by Dunnett's post hoc test was used for statistical analysis. *$p \leq 0.05$, **$p \leq 0.01$, ***$p \leq 0.001$, ****$p \leq 0.0001$. 3′ETS, 3′ external transcribed spacer; 5′ETS, 5′ external transcribed spacer; ITS1, internal transcribed spacer 1; ITS2, internal transcribed spacer 2; n.s., not significant.

ribosome assembly.[3,23] A significant increase in the nucleolar size and additional nucleolar dysfunctions have been reported in motor neurons of a spinal muscular atrophy mouse model that were associated with disturbances in snoRNP biogenesis and rRNA processing.[32] Further evidence for an impaired pre-rRNA processing in the fibroblasts with the homozygous *NOP58* variant was obtained from our RNA analyses, as the levels of the 45S and 21S rRNA precursors were significantly increased. An unexpected finding was the almost complete failure to amplify an RT-PCR product with three primer pairs using cDNA derived from proband but not from control fibroblasts, suggesting a specific effect in the proband cells. For cDNA synthesis, we used a retroviral reverse transcriptase, which is known to pause in the presence of secondary structure and/or modified nucleotides in non-coding RNAs, including rRNA. Modifications of rRNA can either permit reverse transcription, induce polymerase pausing, or completely block nucleotide incorporation. A block in polymerization is likely caused by an inability to form a base pair with any canonical nucleotide or by steric hindrance that impedes recognition of the modified residue

by the reverse transcriptase.[33,34] Based on the known limitations of retroviral reverse transcriptases, the inability to amplify RT-PCR products with some primer pairs suggests that pre-rRNAs in proband cells may be hypomethylated, accompanied by secondary structural stabilization, and/or show non-canonical modifications at specific sites that induce polymerase stalling and dissociation from the transcript. Interestingly, the fragile X mental retardation protein (FMRP), loss of which leads to fragile X syndrome in humans, interacts with snoRNAs and contributes to differential rRNA methylation.[35] A trend of rRNA hypermethylation was observed in a human embryonic stem cell line deficient of FMRP,[36] suggesting that alterations in the 2′-*O*-methylation pattern can involve both hypo- and hypermethylation, which collectively contribute to dysregulated protein synthesis and disease.[36–38] Together, data from the literature and our functional data obtained from proband-derived fibroblasts suggest the existence of defects in pre-rRNA methylation and maturation in cells and tissues of the proband with the homozygous synonymous *NOP58* variant.

PEHO syndrome, caused by bi-allelic *ZNHIT3* variants, and the phenotype observed in the proband with the homozygous *NOP58* variant share several clinical features, including microcephaly, global developmental delay, early-onset seizures, and optic atrophy.[14] These findings suggest that defects in box C/D snoRNP biogenesis may particularly impact ribosome function and translation during neuronal development. Recent studies have highlighted the importance of 2′-*O*-methylation in developmental processes.[4] In zebrafish, reduction or loss of rRNA methylation causes profound developmental defects, particularly of the head and brain, and leads to embryonic lethality.[39] Studies in mouse, frog, and human identified differentially modified 2′-*O*-methylation sites in the rRNA during development and across tissues.[4] Specifically, differential rRNA 2′-*O*-methylation defines early stages of development and rRNA 2′-*O*-methylation patterns differ between brain regions during mouse development and between human embryonic stem cells and neural stem cells.[36,40] Taken together, the data show that dynamic rRNA modifications are functionally relevant during development, particularly in the brain.

In conclusion, we show that the homozygous *NOP58* variant c.516G>A; p.Leu172= causes skipping of exon 7 in the majority of *NOP58* pre-mRNAs, but leaves a small amount of transcripts intact. The hypomorphic nature of the *NOP58* variant allows production of a residual amount of NOP58 wild-type protein. However, steady-state levels of fibrillarin and certain box C/D snoRNAs are significantly reduced in proband fibroblasts. Our data provide evidence of impaired pre-rRNA maturation and indirect evidence for an altered 2′-*O*-methylation pattern of rRNAs in the proband's fibroblasts. These findings, together with changes in the morphology and condensate number of nucleoli in proband cells, suggest impairment of the box C/D snoRNP biogenesis that particularly affected brain and neuronal development. Both the *NOP58*-associated neurodevelopmental disorder and PEHO syndrome likely belong to the group of ribosome biogenesis disorders, also known as ribosomopathies, that includes a large spectrum of phenotypes, such as Diamond Blackfan anemia, Treacher-Collins syndrome, dyskeratosis congenita, and hydrops fetalis.[41,42]

Data and code availability

- The published article includes all data generated or analyzed during this study.
- Consent restrictions preclude sharing of full datasets, and the consents do not cover the deposition of the exome sequencing data in a public database. *NOP58* variant and phenotypic information were submitted to the LOVD database (https://databases.lovd.nl/shared/genes/NOP58), with the LOVD Variant ID: 0001045374 and Phenotype ID: 0000351322.

Acknowledgments

We are grateful to the proband's parents who agreed to participate in this project. We thank Sina Ramcke for skillful technical assistance and the UKE Microscopy Imaging Facility (UMIF) at the University Medical Center Hamburg-Eppendorf for technical support. This work was supported by the Deutsche Forschungsgemeinschaft (KU 1240/17-1 to K.K.) and the "Close the Gap" project from the Gender Equality Unit of the University Medical Center Hamburg-Eppendorf.

Declaration of interests

The authors declare no competing interests.

Supplemental information

Supplemental information can be found online at https://doi.org/10.1016/j.xhgg.2025.100557.

Web resources

GeneMatcher, https://genematcher.org/gnomAD: https://gnomad.broadinstitute.org

Online Mendelian Inheritance in Man (OMIM), http://www.omim.org/

Regeneron Genetics Center Million Exome Variant Browser, https://rgc-research.regeneron.com/me/home

Received: June 24, 2025
Accepted: December 8, 2025

References

1. Teixeira, F.K., and Lehmann, R. (2019). Translational Control during Developmental Transitions. Cold Spring Harb. Perspect. Biol. *11*, a032987. https://doi.org/10.1101/cshperspect.a032987.
2. Dorner, K., Ruggeri, C., Zemp, I., and Kutay, U. (2023). Ribosome biogenesis factors-from names to functions. EMBO J. *42*, e112699. https://doi.org/10.15252/embj.2022112699.

3. Vanden Broeck, A., and Klinge, S. (2024). Eukaryotic Ribosome Assembly. Annu. Rev. Biochem. *93*, 189–210. https://doi.org/10.1146/annurev-biochem-030222-113611.
4. Milenkovic, I., and Novoa, E.M. (2025). Dynamic rRNA modifications as a source of ribosome heterogeneity. Trends Cell Biol. *35*, 604–614. https://doi.org/10.1016/j.tcb.2024.10.001.
5. Sloan, K.E., Warda, A.S., Sharma, S., Entian, K.D., Lafontaine, D.L.J., and Bohnsack, M.T. (2017). Tuning the ribosome: The influence of rRNA modification on eukaryotic ribosome biogenesis and function. RNA Biol. *14*, 1138–1152. https://doi.org/10.1080/15476286.2016.1259781.
6. Falaleeva, M., Welden, J.R., Duncan, M.J., and Stamm, S. (2017). C/D-box snoRNAs form methylating and non-methylating ribonucleoprotein complexes: Old dogs show new tricks. Bioessays *39*, 1600264. https://doi.org/10.1002/bies.201600264.
7. Massenet, S., Bertrand, E., and Verheggen, C. (2017). Assembly and trafficking of box C/D and H/ACA snoRNPs. RNA Biol. *14*, 680–692. https://doi.org/10.1080/15476286.2016.1243646.
8. Webster, S.F., and Ghalei, H. (2023). Maturation of small nucleolar RNAs: from production to function. RNA Biol. *20*, 715–736. https://doi.org/10.1080/15476286.2023.2254540.
9. Lafontaine, D.L., and Tollervey, D. (1999). Nop58p is a common component of the box C+D snoRNPs that is required for snoRNA stability. RNA *5*, 455–467. https://doi.org/10.1017/s135583829998192x.
10. Lin, J., Lai, S., Jia, R., Xu, A., Zhang, L., Lu, J., and Ye, K. (2011). Structural basis for site-specific ribose methylation by box C/D RNA protein complexes. Nature *469*, 559–563. https://doi.org/10.1038/nature09688.
11. Watkins, N.J., Ségault, V., Charpentier, B., Nottrott, S., Fabrizio, P., Bachi, A., Wilm, M., Rosbash, M., Branlant, C., and Lührmann, R. (2000). A common core RNP structure shared between the small nucleoar box C/D RNPs and the spliceosomal U4 snRNP. Cell *103*, 457–466. https://doi.org/10.1016/s0092-8674(00)00137-9.
12. Jansen, R.P., Hurt, E.C., Kern, H., Lehtonen, H., Carmo-Fonseca, M., Lapeyre, B., and Tollervey, D. (1991). Evolutionary conservation of the human nucleolar protein fibrillarin and its functional expression in yeast. J. Cell Biol. *113*, 715–729. https://doi.org/10.1083/jcb.113.4.715.
13. Kos, M., and Tollervey, D. (2010). Yeast pre-rRNA processing and modification occur cotranscriptionally. Mol. Cell *37*, 809–820. https://doi.org/10.1016/j.molcel.2010.02.024.
14. Anttonen, A.K., Laari, A., Kousi, M., Yang, Y.J., Jääskeläinen, T., Somer, M., Siintola, E., Jakkula, E., Muona, M., Tegelberg, S., et al. (2017). ZNHIT3 is defective in PEHO syndrome, a severe encephalopathy with cerebellar granule neuron loss. Brain *140*, 1267–1279. https://doi.org/10.1093/brain/awx040.
15. Kobayashi, H., Abe, K., Matsuura, T., Ikeda, Y., Hitomi, T., Akechi, Y., Habu, T., Liu, W., Okuda, H., and Koizumi, A. (2011). Expansion of intronic GGCCTG hexanucleotide repeat in NOP56 causes SCA36, a type of spinocerebellar ataxia accompanied by motor neuron involvement. Am. J. Hum. Genet. *89*, 121–130. https://doi.org/10.1016/j.ajhg.2011.05.015.
16. Ounap, K., Muru, K., Oiglane-Shlik, E., Ilves, P., Pajusalu, S., Kuus, I., Wojcik, M.H., and Reimand, T. (2020). PEHO syndrome caused by compound heterozygote variants in ZNHIT3 gene. Eur. J. Med. Genet. *63*, 103660. https://doi.org/10.1016/j.ejmg.2019.04.017.
17. Rahman, M.L., Bonnard, A.A., Wang, F., Ruaud, L., Guimiot, F., Li, Y., Defer, I., Wang, Y., Marchand, V., Motorin, Y., et al. (2024). New ZNHIT3 Variants Disrupting snoRNP Assembly Cause Prenatal PEHO Syndrome with Isolated Hydrops. Preprint at medRxiv. https://doi.org/10.1101/2024.08.26.24312490.
18. Dreggors-Walker, R.E., Cohen, L.N., Khoshnevis, S., Marchand, V., Motorin, Y., and Ghalei, H. (2022). Studies of mutations of assembly factor Hit1 in budding yeast suggest translation defects as the molecular basis for PEHO syndrome. J. Biol. Chem. *298*, 102261. https://doi.org/10.1016/j.jbc.2022.102261.
19. Chen, S., Francioli, L.C., Goodrich, J.K., Collins, R.L., Kanai, M., Wang, Q., Alföldi, J., Watts, N.A., Vittal, C., Gauthier, L.D., et al. (2024). A genomic mutational constraint map using variation in 76,156 human genomes. Nature *625*, 92–100. https://doi.org/10.1038/s41586-023-06045-0.
20. Sun, K.Y., Bai, X., Chen, S., Bao, S., Zhang, C., Kapoor, M., Backman, J., Joseph, T., Maxwell, E., Mitra, G., et al. (2024). A deep catalogue of protein-coding variation in 983,578 individuals. Nature *631*, 583–592. https://doi.org/10.1038/s41586-024-07556-0.
21. Cartegni, L., Wang, J., Zhu, Z., Zhang, M.Q., and Krainer, A.R. (2003). ESEfinder: A web resource to identify exonic splicing enhancers. Nucleic Acids Res. *31*, 3568–3571. https://doi.org/10.1093/nar/gkg616.
22. Sobreira, N., Schiettecatte, F., Boehm, C., Valle, D., and Hamosh, A. (2015). New tools for Mendelian disease gene identification: PhenoDB variant analysis module; and GeneMatcher, a web-based tool for linking investigators with an interest in the same gene. Hum. Mutat. *36*, 425–431. https://doi.org/10.1002/humu.22769.
23. Lafontaine, D.L.J., Riback, J.A., Bascetin, R., and Brangwynne, C.P. (2021). The nucleolus as a multiphase liquid condensate. Nat. Rev. Mol. Cell Biol. *22*, 165–182. https://doi.org/10.1038/s41580-020-0272-6.
24. Verheggen, C., Mouaikel, J., Thiry, M., Blanchard, J.M., Tollervey, D., Bordonné, R., Lafontaine, D.L., and Bertrand, E. (2001). Box C/D small nucleolar RNA trafficking involves small nucleolar RNP proteins, nucleolar factors and a novel nuclear domain. EMBO J. *20*, 5480–5490. https://doi.org/10.1093/emboj/20.19.5480.
25. Dieci, G., Preti, M., and Montanini, B. (2009). Eukaryotic snoRNAs: a paradigm for gene expression flexibility. Genomics *94*, 83–88. https://doi.org/10.1016/j.ygeno.2009.05.002.
26. Huttenhofer, A., Kiefmann, M., Meier-Ewert, S., O'Brien, J., Lehrach, H., Bachellerie, J.P., and Brosius, J. (2001). RNomics: an experimental approach that identifies 201 candidates for novel, small, non-messenger RNAs in mouse. EMBO J. *20*, 2943–2953. https://doi.org/10.1093/emboj/20.11.2943.
27. Dunbar, D.A., and Baserga, S.J. (1998). The U14 snoRNA is required for 2'-O-methylation of the pre-18S rRNA in Xenopus oocytes. RNA 4, 195–204.
28. Lange, T.S., Borovjagin, A., Maxwell, E.S., and Gerbi, S.A. (1998). Conserved boxes C and D are essential nucleolar localization elements of U14 and U8 snoRNAs. EMBO J. *17*, 3176–3187. https://doi.org/10.1093/emboj/17.11.3176.
29. Mullineux, S.T., and Lafontaine, D.L.J. (2012). Mapping the cleavage sites on mammalian pre-rRNAs: where do we stand?

Biochimie *94*, 1521–1532. https://doi.org/10.1016/j.biochi.2012.02.001.

30. Kiss, A.M., Jády, B.E., Bertrand, E., and Kiss, T. (2004). Human box H/ACA pseudouridylation guide RNA machinery. Mol. Cell Biol. *24*, 5797–5807. https://doi.org/10.1128/MCB.24.13.5797-5807.2004.
31. Gautier, T., Bergès, T., Tollervey, D., and Hurt, E. (1997). Nucleolar KKE/D repeat proteins Nop56p and Nop58p interact with Nop1p and are required for ribosome biogenesis. Mol. Cell Biol. *17*, 7088–7098. https://doi.org/10.1128/MCB.17.12.7088.
32. Tapia, O., Narcís, J.O., Riancho, J., Tarabal, O., Piedrafita, L., Calderó, J., Berciano, M.T., and Lafarga, M. (2017). Cellular bases of the RNA metabolism dysfunction in motor neurons of a murine model of spinal muscular atrophy: Role of Cajal bodies and the nucleolus. Neurobiol. Dis. *108*, 83–99. https://doi.org/10.1016/j.nbd.2017.08.004.
33. Hammerling, M.J., Yoesep, D.J., and Jewett, M.C. (2020). Single enzyme RT-PCR of full-length ribosomal RNA. Synth. Biol. *5*, ysaa028. https://doi.org/10.1093/synbio/ysaa028.
34. Motorin, Y., Muller, S., Behm-Ansmant, I., and Branlant, C. (2007). Identification of modified residues in RNAs by reverse transcription-based methods. Methods Enzymol. *425*, 21–53. https://doi.org/10.1016/S0076-6879(07)25002-5.
35. D'Souza, M.N., Gowda, N.K.C., Tiwari, V., Babu, R.O., Anand, P., Dastidar, S.G., Singh, R., James, O.G., Selvaraj, B., Pal, R., et al. (2018). FMRP Interacts with C/D Box snoRNA in the Nucleus and Regulates Ribosomal RNA Methylation. iScience *9*, 399–411. https://doi.org/10.1016/j.isci.2018.11.007.
36. Ninochka D'Souza, M., Gowda, N.K.C., Hariharan, N., Qadri, S.W., Palakodeti, D., and Muddashetty, R.S. (2025). Altering rRNA 2'O-methylation pattern during neuronal differentiation is regulated by FMRP. RNA Biol. *22*, 1–22. https://doi.org/10.1080/15476286.2025.2563986.
37. Liu, B., Liang, X.H., Piekna-Przybylska, D., Liu, Q., and Fournier, M.J. (2008). Mis-targeted methylation in rRNA can severely impair ribosome synthesis and activity. RNA Biol. *5*, 249–254. https://doi.org/10.4161/rna.6916.
38. Jaafar, M., Paraqindes, H., Gabut, M., Diaz, J.J., Marcel, V., and Durand, S. (2021). 2'O-Ribose Methylation of Ribosomal RNAs: Natural Diversity in Living Organisms, Biological Processes, and Diseases. Cells *10*, 1948. https://doi.org/10.3390/cells10081948.
39. Higa-Nakamine, S., Suzuki, T., Uechi, T., Chakraborty, A., Nakajima, Y., Nakamura, M., Hirano, N., Suzuki, T., and Kenmochi, N. (2012). Loss of ribosomal RNA modification causes developmental defects in zebrafish. Nucleic Acids Res. *40*, 391–398. https://doi.org/10.1093/nar/gkr700.
40. Hafner, S.J., Jansson, M.D., Altinel, K., Andersen, K.L., Abay-Norgaard, Z., Menard, P., Fontenas, M., Sorensen, D.M., Gay, D.M., Arendrup, F.S., et al. (2023). Ribosomal RNA 2'-O-methylation dynamics impact cell fate decisions. Dev. Cell *58*, 1593–1609.e9. https://doi.org/10.1016/j.devcel.2023.06.007.
41. Landry-Voyer, A.M., Holling, T., Mis, E.K., Mir Hassani, Z., Alawi, M., Ji, W., Jeffries, L., Kutsche, K., Bachand, F., and Lakhani, S.A. (2025). Biallelic variants in the conserved ribosomal protein chaperone gene PDCD2 are associated with hydrops fetalis and early pregnancy loss. Proc. Natl. Acad. Sci. USA *122*, e2426078122. https://doi.org/10.1073/pnas.2426078122.
42. Venturi, G., and Montanaro, L. (2020). How Altered Ribosome Production Can Cause or Contribute to Human Disease: The Spectrum of Ribosomopathies. Cells *9*, 2300. https://doi.org/10.3390/cells9102300.

HGGA, Volume ■■

Supplemental information

A homozygous synonymous *NOP58* variant causes a neurodevelopmental disorder by impairing maturation of pre-ribosomal RNAs

Loisa D. Bonde, Tess Holling, Malik Alawi, Ahmed A. El Beheiry, Zabih Mir Hassani, François Bachand, Ibrahim M. Abdelrazek, and Kerstin Kutsche

SUPPLEMENTAL INFORMATION

SUPPLEMENTAL FIGURES AND LEGENDS

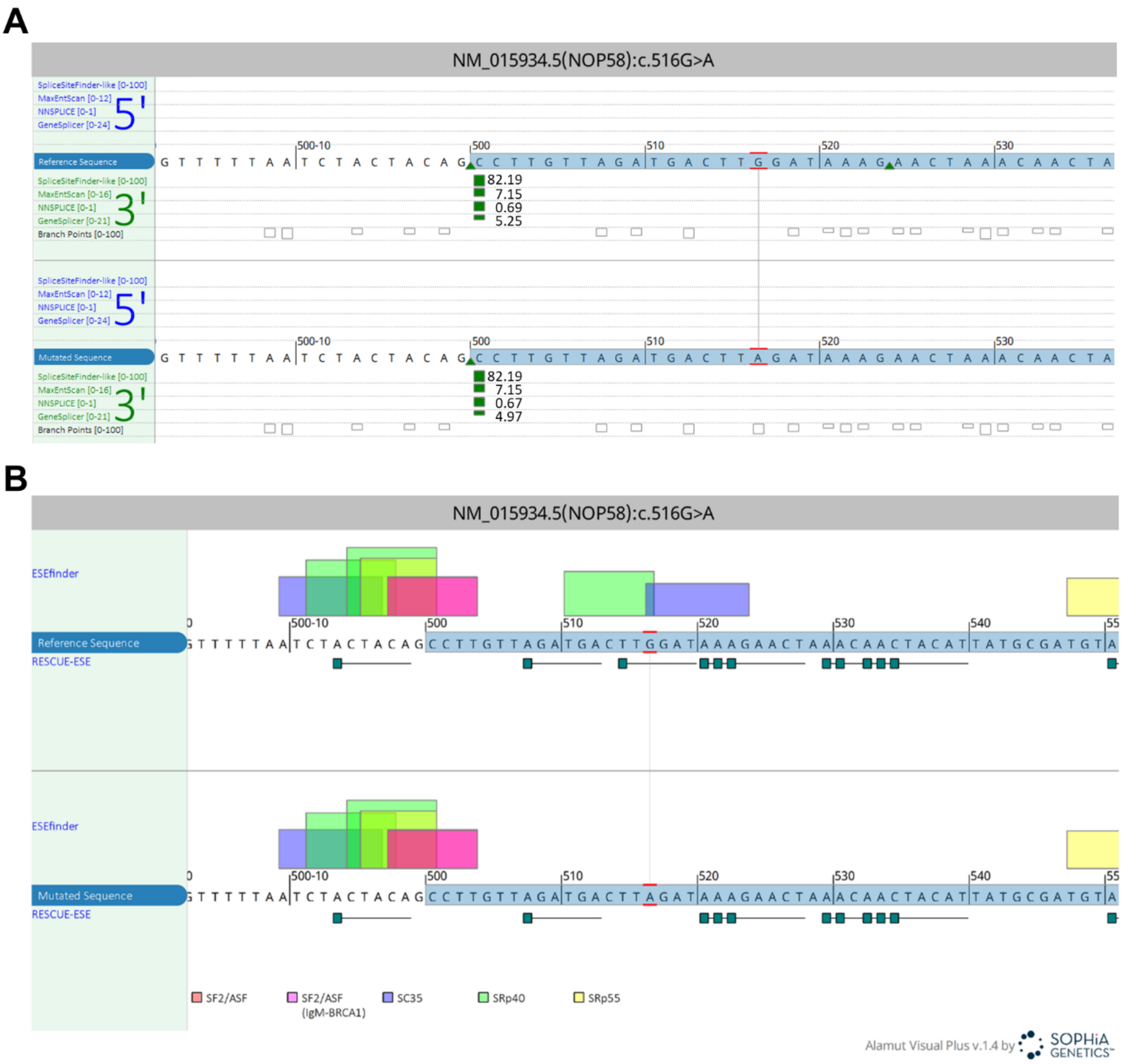

Figure S1. Splice site and exonic splicing enhancer predictions for the homozygous synonymous variant c.516G>A in *NOP58.*

(A-B) Screenshots from the splicing module of Alamut Visual Plus (v1.4; SOPHIA GENETICS) show partial sequences of the intron 6-exon 7 boundary of *NOP58* (NM_015934.5). The reference sequence is displayed in the upper panel and the sequence with the variant c.516G>A in the lower panel. The exonic sequence is highlighted in blue. **(A)** Splice sites were predicted using the programs SpliceSiteFinder-like,[1,2] NNSPLICE 0.9 version,[3] MaxEntScan,[4] and GeneSplicer.[5] The predicted splice acceptor sites are indicated by a green rectangle within the sequence; scores are given (see also Table S2) and indicated as green boxes below the first nucleotide of exon 7. **(B)** Exonic splicing enhancers (ESE) were predicted using the programs ESEfinder[6] and RESCUE-ESE.[7] ESE hits from ESEfinder are displayed as colored boxes above each sequence. The height of each box indicates the score value, while its width and position show the length and position of the motif in the sequence. Predicted RESCUE-ESE hexamers are shown beneath each sequence as petrol boxes, with a line indicating the length of the motif.

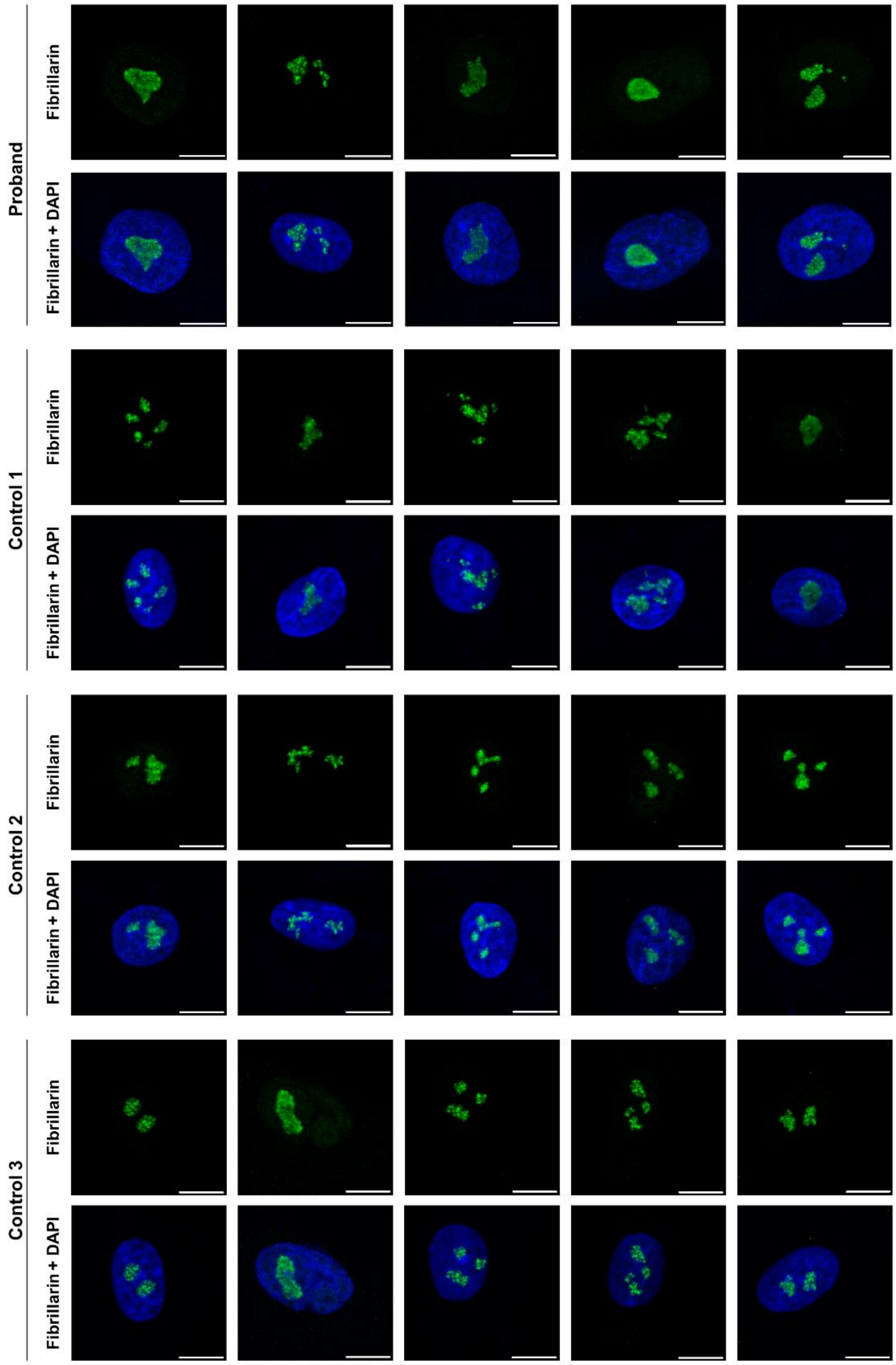

Figure S2. Representative immunofluorescence images of fibrillarin staining in proband and control cells.

Immunofluorescence analysis using an anti-fibrillarin antibody followed by anti-mouse Alexa Fluor 488-conjugated secondary antibody (green) to stain nucleoli in proband and control fibroblasts. Nuclear DNA was stained with DAPI (blue). Fibroblasts were seeded on coverslips and cultivated under basal conditions. Cells were imaged by confocal fluorescence microscopy. Representative images of fibroblasts of the proband (top) and fibroblasts from three controls are shown. Scale bar, 10 µm.

SUPPLEMENTAL TABLES

Table S1. Sequence of oligonucleotides used in this work.

***NOP58* primer sequences for variant validation**

Template	Exon	Direction	Sequence (5' → 3')
DNA	7	forward	TGCCTGTCTGCTGGGATTAC
		reverse	CAAAAACCCTGGGAATCTTGC

***NOP58* primer sequences for RT-PCR**

Template	Name	Exon	Direction	Sequence (5' → 3')
cDNA	c5F	5	forward	CGTGAAATGGCAGCTATGTGTC
	c8R	8	reverse	TCAGAAAGCTTGGCAGAGGC
	c7R	7	reverse	TCCTAATTCAGGGAAATGCCAGCC

***NOP58* primer sequences for RT-qPCR**

Template	Name	Exon	Direction	Sequence (5' → 3')
cDNA	5F	5	forward	CTCAGTTGTATCCATAGTCC
	6R	6	reverse	CTTTATCAGCGCTAAACTTC
	11F	11	forward	GAGAACTTTGGAAGACAGAG
	13R	13	reverse	TTTAGAACAGGTTGGAAGTG
	6F	6	forward	CGCTGATAAAGTAGACACAA
	7R	7	reverse	TTCCTAATTCAGGGAAATGC
	7F	7	forward	CTACATTATGCGATGTAGAG
	8R	8	reverse	GAGGCATAGTTCTTCCTATC

snoRNA primer sequences for RT-qPCR

Template	Target	Direction	Sequence (5' → 3')
cDNA	*SNORD14A* (*U14A*) NR_000022.1	forward	GATTGGTTGCCAGACATTCG
		reverse	CACTCAGACATCCAAGGAAG
	SNORD93 (*HBII-336*) NR_003075.1	forward	GCCAAGGATGAGAACTCTAATCTGA
		reverse	GGCCTCAGGTAAATCCTTTAATCCA
	SNORD125 NR_003686.1	forward	CCCTCCTGATGATTCTTCTTCC
		reverse	GTCAACTTCTTAGAGGCTCAGTT
	SNORD18A (*U18A*) NR_002441.1	forward	TAGTGATGAAATTCCACTTC
		reverse	CATCAGAACATCCGAGAAAA
	SNORD91B (*HBII-296B*) NR_003073.1	forward	GTCTGAACCTGTCTGAAGCATCC
		reverse	AAGCCTCAGTATCACACAGAAGT
	SNORD3A (*U3*) NR_006880.1	forward	TAGCAGAGGTGTGCAAGGAGG
		reverse	GCAGTTGCAGCCAAGCAACG
	SNORA30 (*ACA30*) NR_002966.1	forward	GCACTTTCACAGTTCCTTCC
		reverse	CAGGGCAAGAATACAATCAAGG

rRNA primer sequences for RT-qPCR

cDNA	r01-F	forward	CGTGCGTGTCAGGCGTTCTC
	r01-R	reverse	GGTCCCGTTCGCCACGAAC
	r02-F	forward	CAGAGCAGCTCCCTCGCTG
	r02-R	reverse	GGAGGCGGGAACCGAAGAAG
	r2-F	forward	GTGGGGGCTTTACCCGGC
	r2-R	reverse	CCCAAGAGGAGAGGGGGTTG
	r3'-F	forward	CTCTCCCGTCGCCTCTCC
	r3'-R	reverse	CAGAGCAGCTCCCTCGCTG
	rA0-F	forward	GGGGGAGAAGCGAGGGTTCC
	rA0-R	reverse	AGTCACGCGCCGGACAGAG
	r1-F	forward	CCGTCGTCCTCCTCGCTTG

	r1-R	reverse	GAGCGAGCGACCAAAGGAACC
	rC-F	forward	GAGCGAGCGACCAAAGGAACC
cDNA	rC-R	reverse	GCCGGGTAAAGCCCCCAC
	r18S-F	forward	GTCCCTGCCCTTTGTACACAC
	rE-R	reverse	CCGTCTCCCTCCCGAGTTC
	r3-R	reverse	CCTCCGGGCTCCGTTAATG
	r18S-total-R	reverse	GATCCTTCCGCAGGTTCACC

Exon numbering is given according to the *NOP58* reference sequence NM_015934.5.

Table S2. *In silico* pathogenicity and splice site predictions and minor allele frequency of the *NOP58* variant.

Genomic position on chromosome 2 (hg38; NC_000002.12)	Nucleotide and predicted amino acid change (NM_015934.5)	Exon	Variant description after transcript analysis	gnomAD (v4.1.0)	RGC Million Exome	CADD (>20)	Splice site predictions							
							SpliceSite Finder-like (range 0-100)		NNSPLICE (range 0-1)		MaxEntScan (range 0-16)		GeneSplicer (range 0-21)	
							WT	Var	WT	Var	WT	Var	WT	Var
g.202290339G>A	c.516G>A; p.Leu172=	7	r.500_634del; p.(Ser167_Gly212delinsCys)	absent	absent	17.25	82.19	82.19	0.69	0.67	7.15	7.15	5.25	4.97

Worldwide allele frequency of *NOP58* variant in the gnomAD database v4.1.0 [8] and the Regeneron Genetics Center (RGC) Million Exome data[9] is given. The functional impact of the homozygous *NOP58* variants was predicted by the Combined Annotation Dependent Depletion (CADD) tool. CADD is a framework that integrates multiple annotations in one metric by contrasting variants that survived natural selection with simulated mutations. Reported CADD scores are phred-like rank scores based on the rank of that variant's score among all possible single nucleotide variants of hg19, with 10 corresponding to the top 10%, 20 at the top 1%, and 30 at the top 0.1%. The larger the score the more likely the variant has deleterious effects; the score range observed here is strongly supportive of pathogenicity, with all observed variants ranking above ~99% of all variants in a typical genome and scoring similarly to variants reported in ClinVar as pathogenic (~85% of which scores >15).[10] Acceptor splice site prediction scores were predicted for wild-type and mutated sequences using Alamut Visual Plus (v1.4; SOPHIA GENETICS) which implements the following splice site prediction algorithms: SpliceSiteFinder-like,[1,2] NNSPLICE 0.9 version,[3] MaxEntScan,[4] and GeneSplicer.[5] High scores indicate strong splice sites. Var, variant; WT, wildtype.

SUPPLEMENTAL METHODS

Trio whole-exome sequencing and variant filtering

Genomic DNA was extracted from leukocytes of the proband and his parents by standard procedures. Trio whole-exome sequencing (WES) was performed on genomic DNA samples of the proband and his parents. Exome sequencing libraries were generated using the Twist Human Core Exome Plus kit (Twist Bioscience). Libraries were sequenced on an Illumina platform by CeGaT (Tübingen, Germany). fastp[11] (v0.21.0) was used to remove artificial and low quality (Phred quality score below 15) sequences. Putative base calling errors located in regions where two reads of a read pair overlap were corrected (fastp option: '--correction'). The sequences were then aligned to the human reference assembly [NCBI GRCh38 (GCA_000001405.15)] with the Burrows-Wheeler Aligner (BWA mem, v0.7.17-r1188).[12] Strelka2 (v2.9.10)[13] and GATK4 (v4.1.9.0)[14] were used to detect genetic variation. Variants were annotated using the Ensembl Variant Effect Predictor (v103.0).[15]

The trio exome data were analyzed with respect to (i) *de novo* variants (allele frequency ≤0.5%), which are present in the proband and absent in both parents, (ii) autosomal recessive variants (allele frequency ≤0.5%), which are present in the compound heterozygous or homozygous state in the proband and heterozygous in the parents, (iii) X-linked variants (allele frequency ≤0.5%), which are present in the hemizygous state in the proband and inherited from the mother, and (iv) copy number variations (CNVs) of exonic and surrounding non-coding regions. Sequence variants are described according to the nomenclature of the Human Genome Variation Society (HGVS nomenclature). Variant prioritization was based on database queries and *in silico* pathogenicity programs (AlphaMissense,[16] CADD,[10] and REVEL[17]), splice site predictions (Alamut Visual Plus v1.4; SOPHIA GENETICS, providing: GeneSplicer,[5] MaxEntScan,[4] NNSplice 0.9,[3] and SpliceSiteFinder-like[2]), and exonic splicing enhancer (ESE) predictions (Alamut Visual Plus v1.4; SOPHIA GENETICS, providing: ESEfinder and RESCUE-ESE).[6,7]

Variant validation and segregation

Sanger sequencing permitted *NOP58* (NM_015934.5) variant validation and/or segregation in leukocyte-derived DNA from the proband, his healthy sister, and parents. PCR amplicons were generated according to standard PCR protocols with the OneTaq® Quick-Load 2 × Master Mix (New England Biolabs). Primers were designed to amplify the variant-containing exon 7 of *NOP58* (NM_015934.5) and adjacent intronic sequences (**Table S1**). Amplicons were directly sequenced using the ABI BigDye Terminator Sequencing Kit (Applied Biosystems) and an automated capillary sequencer (ABI 3500, Applied Biosystems). Sequence electropherograms were analyzed using SeqManPro™ (DNASTAR® Software for Life Scientists) and Chromas Lite 2.1.1 (Technelysium Pty Ltd). The *NOP58* variant was described according to the GenBank reference sequences NM_015934.5 and NP_057018.1. Correct variant nomenclature was assessed using Mutalyzer (https://mutalyzer.nl/name-checker).

Cell culture

Primary dermal fibroblasts were cultured from a skin biopsy of the proband and three healthy female controls (Ctrl. 1-3, all 4 years old) in Dulbecco's modified Eagle medium (DMEM; Thermo Fisher Scientific) supplemented with 10% fetal bovine serum (FBS; GE Healthcare) and penicillin-streptomycin (100 U/mL and 100 mg/mL, respectively; Thermo Fisher Scientific). The same passage number of proband and control fibroblasts was used in all experiments. Primary fibroblasts were regularly tested for mycoplasma contamination and confirmed to be mycoplasma-free.

RNA isolation and transcript analysis

300,000 primary fibroblasts from the proband and controls were seeded into 6 cm culture dishes. The following day, total RNA was extracted using the Monarch Total RNA Miniprep Kit (New England Biolabs). The RNA concentration and purity of the samples were assessed using the Epoch™ Microplate Spectrophotometer (Biotek Instruments). 1 µg of total RNA was reverse transcribed using the LunaScript®RT Super Mix Kit (New England Biolabs). Reverse transcription (RT)-PCR fragments were

generated according to standard PCR protocols with OneTaq® Quick-Load® 2× Master Mix (New England Biolabs). Primer sequences are described in **Table S1**. RT-PCR products were directly Sanger-sequenced.

Real-time quantitative PCR (RT-qPCR)

RT-qPCR was performed to determine relative *NOP58* mRNA, snoRNA, and rRNA levels. Technical triplicates of RT-qPCR samples were prepared as a 10-µL approach using the SYBR Green I- based Luna Universal qPCR Master Mix (New England Biolabs), 500 nM of each primer, and 1 µL of the reverse transcription reaction as described above. Primer sequences for RT-qPCR are described in **Table S1**. RT-qPCR was performed using the QuantStudio 3 Real-Time PCR System equipped with the QuantStudio Design&Analysis Software v1.4.3 (Thermo Fisher Scientific).

For relative *NOP58* mRNA and snoRNA analysis, the PCR conditions consisted of a pre-run at 95°C for 5 minutes, followed by 40 cycles of 30 seconds at 95°C, 30 seconds at 58°C, and 45 seconds at 72°C. The specificity of PCR amplification was determined by melting curve analysis with a range from 60°C to 95°C. The cycle threshold (CT) values of the target RNAs were normalized to the housekeeping mRNA of *GAPDH*. $2^{-\Delta Ct}$ values were calculated individually for each experiment and expressed as a fold change to the mean of the three controls. Canonically spliced *NOP58* mRNAs with exon 7 were targeted by using primers located in exons 6 and 7 and exons 7 and 8. Total *NOP58* mRNA levels were determined using primers located in exons 5 and 6 and exons 11 and 13. The proportion of *NOP58* mRNAs with exon 7 was calculated as the ratio of *NOP58* mRNA with exon 7 to total *NOP58* mRNA, using all respective primer combinations.

For rRNA analysis, the PCR conditions consisted of a pre-run at 95°C for 90 seconds, followed by 40 cycles of 10 seconds at 94°C, 30 seconds at 55°C, and 45 seconds at 72°C. The specificity of PCR amplification was determined by melting curve analysis with a range from 60°C to 95°C. The cycle threshold (CT) values of the target rRNA precursors were normalized to the total 18S rRNA amplified

using primers r18S-F and r18S-total-R (**Table S1**). $2^{-\Delta\Delta Ct}$ values were calculated individually for each experiment and expressed as a fold change to the mean of the three controls.

Antibodies and reagents

Primary antibodies and dilutions used: rabbit anti-NOP58 antibody (BethylLabs; #A302-719A; WB: 1:1,000 in 5% BSA/TBST), mouse anti-fibrillarin antibody (abcam; #ab18380; WB: 1:500 in 5% mTBST; IF: 1:100), mouse anti-NOP56 antibody (invitrogen; #MA5-24641; WB: 1:500 in 5% BSA/TBST), rabbit anti-SNU13/anti-NHP2L1 antibody (abcam; #ab95958; WB: 1:500 in 5% BSA/TBST), hFAB™ Rhodamine anti-GAPDH antibody (Bio-Rad; #12004167; WB: 1:10,000), and hFAB™ Rhodamine anti-tubulin antibody (Bio-Rad; #12004165; WB: 1:10,000).

Secondary antibodies and dilutions used: goat anti-mouse IgG:StarBright Blue 700 antibody (Bio-Rad; #12004159; WB: 1:7,500-10,000); goat anti-rabbit IgG:StarBright Blue 700 antibody (Bio-Rad; #12004162; WB: 1:7,500-10,000), and goat anti-mouse IgG secondary antibody Alexa Fluor 488-conjugated (Invitrogen; #A11029; IF: 1:1,000).

Reagent used: ProLong™ Diamond Antifade Mountant with DAPI (Invitrogen; #P36962).

Immunoblotting

150,000 fibroblasts were seeded into six-well plates. The following day, cells were harvested in ice-cold RIPA buffer [50 mM Tris-HCl, pH 8.0; 150 mM NaCl; 1% NP-40; 0.5% DOC (sodium deoxycholate); 0.1% SDS (sodium dodecyl sulfate)] supplemented with Mini Protease Inhibitor and PhosSTOP (Roche) and lysed on ice for 10 minutes. Cell debris was removed by centrifugation for 10 min at 4°C, 4x sample buffer (33% glycerol, 80 mM Tris-HCl pH 6.8, 0.3 M DTT, 6.7% SDS, and 0.1% bromophenol blue) was added to the supernatant, and samples were boiled at 95°C for 5 min. Equal amounts of whole-cell lysates were loaded and separated on a Mini PROTEAN® TGX Stain-Free Gel (Bio-Rad) under denaturing conditions, followed by transfer to polyvinylidene fluoride membranes. Membranes were blocked in 5% milk in TBST (mTBST) or 5% BSA in TBST and incubated with the primary antibody overnight in

blocking solution at 4°C. After washing, secondary antibodies were incubated for 1 hour in 0.5% mTBST or 0.5% BSA in TBST at room temperature. Immunoblots were digitally imaged using a ChemiDoc MP (Bio-Rad), with exposure time optimized to avoid saturation. Bands were automatically defined and intensities were determined using the built-in band detection tool of the Image Lab v6.0 software (Bio-Rad). Levels of target proteins were normalized to tubulin or GAPDH.

Immunofluorescence, confocal fluorescence microscopy, and quantitative analyses

30,000 fibroblasts of the proband and three controls were cultivated on glass coverslips in 12-well plates. The following day, cells were fixed with 4% paraformaldehyde (PFA) in phosphate-buffered saline (PBS) and washed three times with PBS. After treatment with permeabilization/blocking solution (2% bovine serum albumin; 3% goat serum; 0.5% Nonidet P40 in PBS), cells were incubated in antibody solution (3% goat serum; 0.1% Nonidet P40 in PBS) containing the primary antibody. Cells were washed with PBS and incubated with Alexa Fluor 488-conjugated secondary antibody. After extensive washing with PBS, cells were embedded in mounting solution with DAPI. Cells were analyzed with Leica TCS SP8 X confocal microscope (Microscope: Leica DMi8; Objective: 63× HC PL APO Oil CS2, NA: 1.4; Software: Leica LAS X SP8). ImageJ (v1.54f; NIH) was used for visualization.

Nucleolar structure was assessed on blinded samples after fibrillarin staining using two different criteria: 1) cells were categorized according to the number of condensates per cells, ranging from one to seven; 2) cells were categorized into two groups: (i) cells with normal nucleolar structure, showing at least one very bright spot within the condensates and (ii) cells with abnormal nucleolar structure, showing fluorescence signals evenly distributed throughout the condensates. Nucleolar structure was assessed in a minimum of 20 cells per fibroblast cell line and experiment.

Data analysis and statistics

Quantitative data are presented by Prism v10.2.3 software (GraphPad Software) as the mean ± standard deviation (SD). For quantification, one- or two-way ANOVA followed by a Dunnett's

post hoc test for multiple comparisons was performed. A $p \leq 0.05$ was considered statistically significant (*$p \leq 0.05$; **$p \leq 0.01$; ***$p \leq 0.001$; ****$p \leq 0.0001$).

SUPPLEMENTAL REFERENCES

1. Zhang, M.Q. (1998). Statistical features of human exons and their flanking regions. Hum Mol Genet *7*, 919-932. 10.1093/hmg/7.5.919.
2. Shapiro, M.B., and Senapathy, P. (1987). RNA splice junctions of different classes of eukaryotes: sequence statistics and functional implications in gene expression. Nucleic Acids Res *15*, 7155-7174. 10.1093/nar/15.17.7155.
3. Reese, M.G., Eeckman, F.H., Kulp, D., and Haussler, D. (1997). Improved splice site detection in Genie. J Comput Biol *4*, 311-323. 10.1089/cmb.1997.4.311.
4. Yeo, G., and Burge, C.B. (2004). Maximum entropy modeling of short sequence motifs with applications to RNA splicing signals. J Comput Biol *11*, 377-394. 10.1089/1066527041410418.
5. Pertea, M., Lin, X., and Salzberg, S.L. (2001). GeneSplicer: a new computational method for splice site prediction. Nucleic Acids Res *29*, 1185-1190. 10.1093/nar/29.5.1185.
6. Cartegni, L., Wang, J., Zhu, Z., Zhang, M.Q., and Krainer, A.R. (2003). ESEfinder: A web resource to identify exonic splicing enhancers. Nucleic Acids Res *31*, 3568-3571. 10.1093/nar/gkg616.
7. Fairbrother, W.G., Yeh, R.F., Sharp, P.A., and Burge, C.B. (2002). Predictive identification of exonic splicing enhancers in human genes. Science *297*, 1007-1013. 10.1126/science.1073774.
8. Chen, S., Francioli, L.C., Goodrich, J.K., Collins, R.L., Kanai, M., Wang, Q., Alfoldi, J., Watts, N.A., Vittal, C., Gauthier, L.D., et al. (2024). A genomic mutational constraint map using variation in 76,156 human genomes. Nature *625*, 92-100. 10.1038/s41586-023-06045-0.
9. Sun, K.Y., Bai, X., Chen, S., Bao, S., Zhang, C., Kapoor, M., Backman, J., Joseph, T., Maxwell, E., Mitra, G., et al. (2024). A deep catalogue of protein-coding variation in 983,578 individuals. Nature *631*, 583-592. 10.1038/s41586-024-07556-0.
10. Kircher, M., Witten, D.M., Jain, P., O'Roak, B.J., Cooper, G.M., and Shendure, J. (2014). A general framework for estimating the relative pathogenicity of human genetic variants. Nat Genet *46*, 310-315. 10.1038/ng.2892.
11. Chen, S., Zhou, Y., Chen, Y., and Gu, J. (2018). fastp: an ultra-fast all-in-one FASTQ preprocessor. Bioinformatics *34*, i884-i890. 10.1093/bioinformatics/bty560.
12. Li, H., and Durbin, R. (2010). Fast and accurate long-read alignment with Burrows-Wheeler transform. Bioinformatics *26*, 589-595. 10.1093/bioinformatics/btp698.
13. Kim, S., Scheffler, K., Halpern, A.L., Bekritsky, M.A., Noh, E., Kallberg, M., Chen, X., Kim, Y., Beyter, D., Krusche, P., and Saunders, C.T. (2018). Strelka2: fast and accurate calling of germline and somatic variants. Nat Methods *15*, 591-594. 10.1038/s41592-018-0051-x.
14. McKenna, A., Hanna, M., Banks, E., Sivachenko, A., Cibulskis, K., Kernytsky, A., Garimella, K., Altshuler, D., Gabriel, S., Daly, M., and DePristo, M.A. (2010). The Genome Analysis Toolkit: a MapReduce framework for analyzing next-generation DNA sequencing data. Genome Res *20*, 1297-1303. 10.1101/gr.107524.110.
15. McLaren, W., Gil, L., Hunt, S.E., Riat, H.S., Ritchie, G.R., Thormann, A., Flicek, P., and Cunningham, F. (2016). The Ensembl Variant Effect Predictor. Genome Biol *17*, 122. 10.1186/s13059-016-0974-4.
16. Cheng, J., Novati, G., Pan, J., Bycroft, C., Zemgulyte, A., Applebaum, T., Pritzel, A., Wong, L.H., Zielinski, M., Sargeant, T., et al. (2023). Accurate proteome-wide missense variant effect prediction with AlphaMissense. Science *381*, eadg7492. 10.1126/science.adg7492.
17. Ioannidis, N.M., Rothstein, J.H., Pejaver, V., Middha, S., McDonnell, S.K., Baheti, S., Musolf, A., Li, Q., Holzinger, E., Karyadi, D., et al. (2016). REVEL: An Ensemble Method for Predicting the Pathogenicity of Rare Missense Variants. Am J Hum Genet *99*, 877-885. 10.1016/j.ajhg.2016.08.016.
